# Nano-Space Confinement Drives Rational Closed Pore Design in Hard Carbons for High-Capacity and High-Rate Sodium Storage

**DOI:** 10.1007/s40820-026-02223-7

**Published:** 2026-05-21

**Authors:** Run Ren, Ling Zhang, Jianhua Zhu, Yunfeng Chao, Junlin Guo, Yijun Cao, Xiaobo Ji, Xinwei Cui

**Affiliations:** 1 Henan Institutes of Advanced Technology, Zhengzhou University, Zhengzhou, 450003 People’s Republic of China; 2 Zhongyuan Critical Metals Laboratory, Zhengzhou University, Zhengzhou, 450001 People’s Republic of China; 3 School of Materials Science and Engineering, Zhengzhou University, Zhengzhou, 450001 People’s Republic of China; 4 College of Chemistry and Chemical Engineering, Central South University, Changsha, 410083 People’s Republic of China; 5 State Key Laboratory of Coking Coal Resources Green Exploitation, Zhengzhou University, Zhengzhou, 450001 People’s Republic of China

**Keywords:** Sodium-ion batteries, Hard carbon, Nanoconfinement, Closed pore, Sodium storage mechanism

## Abstract

**Highlights:**

- Nano-space confinement regulates heterogeneous nucleation of quasi-metallic Na clusters in closed graphitic pores of hard carbons, suggesting a coupled “intercalation-pore filling” and stage-wise storage mechanism for high Na-storage capacities.
- A new stage near the end of slope region was identified, where confined nano-spaces at 0.4–0.6 nm facilitate pre-desolvation and enhance Na-ion transport kinetics for high-rate capabilities.
- Rational design of stage-wise closed pores was achieved in hard carbons, resulting in superior performance of 500 mAh g^−1^ at 50 mA g^−1^ and 344 mAh g^−1^ at 2000 mA g^−1^.

**Abstract:**

Hard carbons are emerging as the most viable anodes for the commercialization of Na-ion batteries. However, their performance limits are far from being disclosed because of ambiguous Na-storage mechanism. Here, we report that nano-space confinement regulates heterogeneous nucleation of quasi-metallic Na clusters in closed pores, uncovering a coupled “intercalation-pore filling” and stage-wise storage mechanism for high capacities. Theoretical studies reveal that the energy barrier for Na-cluster growth decreases as the nanocavity size decreases; however, it remains energetically unfavorable at potentials (V vs. Na/Na^+^) > 0. Interestingly, in the coupled storage, Na-ion intercalation in nanoconfined orifices triggers stepwise pre-nucleation, reducing energy barriers for spontaneous Na-cluster growth in progressively larger cavities at positive potentials, thus enabling Na-cluster deposition into previously unused closed pores. This understanding guides the rational design of stage-wise closed pores, resulting in superior performance of 500 mAh g^−1^ at 50 mA g^−1^ and 344 mAh g^−1^ at 2000 mA g^−1^. Mechanistic studies further identify a new stage, where confined nano-spaces at 0.4–0.6 nm facilitate pre-desolvation and enhance Na-ion transport kinetics for high-rate capabilities. This work identifies the origin governing Na-storage behavior in the closed pores of hard carbons, boosting their overall performance beyond prior expectations.

**Figure:**
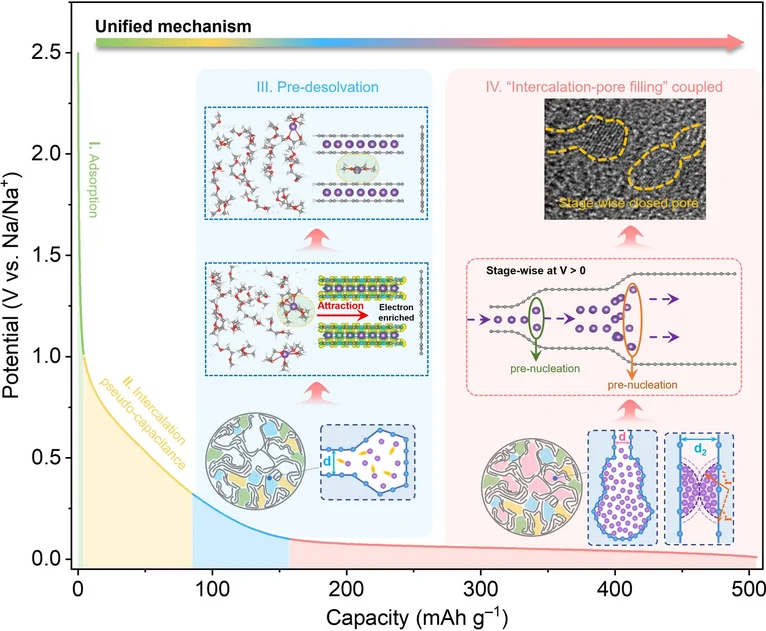

**Supplementary Information:**

The online version contains supplementary material available at 10.1007/s40820-026-02223-7.

## Introduction

The abundance of sodium (Na) reserves in the earth’s crust makes sodium-ion batteries (SIBs) a sustainable and cost-effective alternative to lithium-ion batteries (LIBs) [1–3]. However, limitations in energy and power densities currently restrict SIBs to being positioned as supplementary energy storage systems alongside LIBs [4–6]. On the cathode side, potential materials such as layer oxides (NaNi_1/3_Co_1/3_Mn_1/3_O_2_) [7], polyanion compounds (NaFePO_4_, Na_3_V_2_(PO_4_)_3_) [8, 9], Prussian blue derivatives [10, 11] are constrained by well-defined performance ceilings, specifically their theoretical capacities. In contrast, on the anode side, the performance limits of the most viable anode material, hard carbon (HC), remain unclear [12–14]. Therefore, fully exploiting the potential of HCs presents a promising approach for advancing the development of SIBs. A major hurdle, however, is that HCs are amorphous materials with short-range order, often described by a “house of cards” model [15], making it challenging to establish the relationship between the pore structure of HCs and Na-storage behavior.

Massive efforts have been devoted to clarify the Na storage mechanism in HCs [16–18]. Despite ongoing debates [19–21], there is a general consensus that the Na storage in HC basically follows “adsorption-intercalation/filling” mechanism [22]. The uptake of Na ions in HCs begins with their adsorption at defective sites and the surfaces of open pores, leading to a slope region in the high-potential range between 1.0 and 0.1 V [23–25]. Following this, intercalation of Na ions between graphitic layers and pore filling of quasi-metallic Na clusters into closed pores occur, either separately or concurrently, contributing to the low-potential plateau region below 0.1 V [26, 27]. The plateau capacity typically accounts for 60%–90% of the total capacity, depending on the size and geometry of the closed pores [28–30]. Thus, closed pores play a critical role in determining the energy density for practical applications. However, it has been reported that only 60% of the closed pores are filled after sodiation, indicating the substantial room for further improving the Na-storage capacity [31, 32]. Moreover, it is generally believed that increasing the capacity contribution of the plateau region from the closed pores could reduce the rate capability [33, 34]. Therefore, finding strategies to access the unused closed pores without compromising rate performance is essential for maximizing Na-storage performance in HCs. However, the consensual mechanisms do not provide a clear solution.

In previous studies, the Na storage mechanism was addressed only through the detected form that Na adopts within the pores (i.e., adsorbed/intercalated ions or quasi-metals). However, the critical question remains: what governs the form of Na in the pores? Moreover, electrodeposition of bulk Na metal occurs at potentials (V vs. Na/Na⁺) < 0, whereas the deposition of quasi-metallic Na clusters within the closed pores takes place at potentials > 0. What drives this potential upshift? By correctly addressing the underlying mechanism, the relationship between the pore structure of HCs and Na-storage behavior can be analyzed, offering guidance for maximizing Na-storage performance. A key observation is that the electrochemical behaviors of Li and Na deposition differ significantly on the surface of graphitic carbons [35, 36]. It inspires us that the interaction between Na and the graphitic walls may be the fundamental factor determining the distinct storage behaviors. The question thus arises as to the mechanism by which closed pores modulate those interfacial interactions.

Herein, we report that nano-space confinement is the origin governing interfacial interactions within the orifices and cavities of closed graphitic pores, which ultimately raises the deposition potential of quasi-metallic Na clusters above zero and drives highly efficient Na storage in HCs. Deriving from heterogeneous nucleation in confined nano-space, a coupled “intercalation-pore filling” storage mechanism is suggested for high capacities. It is revealed that an orifice with highly confined interlayer spacings permits Na ions to intercalate at the onset of the plateau region. With appropriate control of closed pore size and geometry, this intercalation can subsequently induce stepwise pre-nucleation for the spontaneous Na-cluster growth in progressively larger cavities at positive potentials, thus capable of accessing previously unused closed pores for high Na-storage capacities. Furthermore, mechanistic studies indicate that the confined nano-spaces between 0.4 and 0.6 nm not only facilitate pre-desolvation but also improve Na-ion transport kinetics near the end of the slope region, determining high-rate performance. All these insights have been validated by atomic-level imaging, in situ spectroscopic analysis, and density-functional-theory (DFT) calculations. Accordingly, a type of stage-wise closed graphitic pores with the optimal orifice and cavity sizes was realized experimentally, achieving superior Na-storage performance in both half cells and full cells. This work may provide universal design principles for HCs to promote the sustainable development of high-performance Na-ion batteries.

## Experimental Section

### Materials

Hexamethylenetetramine (C_6_H_12_N_4_, HMTA), resorcinol (C_6_H_6_O_2_), and ethanol were purchased from Sigma-Aldrich. The electrolyte was purchased from Duoduo Reagent Company. Milli-Q water (18 MΩ) was made by PUXI GWB-2. All of the analytical reagents mentioned above were used without further purification. A glass Petri dish with a diameter of 9 cm and a height of 1.6 cm was used. Metallic sodium disks, CR20232 battery case components, the separator of glass fibers (Whatman GF/D) and Polypropylene (PP) membranes were purchased from Kelude Company.

### Synthesis of A-, B-, C-Stage Phenolic Resins

After mixing 0.432 g of resorcinol with 1.160 g of HMTA, the mixture was dissolved in 10 mL of a 7:2 (v/v) ethanol–water solution under continuous stirring for 60 min to yield a clear transparent solution. The solution was then transferred into Petri dishes and aged for 12, 48, and 120 h at room temperature to obtain the A-stage, B-stage, and C-stage prepolymers, respectively. Afterward, the aged prepolymer solutions were thermo-polymerized at 80 °C for 12 h to obtain the A-stage, B-stage, and C-stage phenolic resins.

### Synthesis of Hard Carbons Using B-staged Resin

The B-stage resin was ground, and the resulting powders were subjected to a two-step carbonization process in a high-temperature furnace under an Ar atmosphere (60 ccm). The process consisted of: (1) heating to 800 °C at a rate of 5 °C min^−1^ and holding for 30 min for pre-carbonization; and (2) further heating to 1100, 1300, or 1500 °C at 2 °C min^−1^, followed by a 2h hold for complete carbonization. The furnace was then cooled to 800 °C at 2 °C min^−1^, further to 500 °C at 5 °C min^−1^, and finally allowed to cool naturally to room temperature.

The preparation methods for HCs from A- and C-stage resins remain the same as the aforementioned method, except that the maximum pyrolysis temperature was adjusted to 1300 °C only (A- and C-stage resin-derived HCs).

### Characterizations

Transmission electron microscopy (TEM) analysis was performed using an FEI Tecnai F20 instrument operated at 200 kV. The porous texture characterization was conducted through a combination of: (i) gas physisorption measurements (ASAP 2460 analyzer) with N_2_ at 77 K and CO_2_ at 273.2 K, (ii) true density determination via helium pycnometry (AccuPyc II 1340), and (iii) small-angle X-ray scattering (SAXS) experiments (Xeuss 3.0 system). X-ray diffraction (XRD) patterns were recorded on a Bruker D8 Advance diffractometer equipped with Cu Kα radiation (*λ* = 1.54 Å) in Bragg–Brentano geometry. Fourier-transform infrared (FTIR) spectra were acquired using a Bruker VERTEX 70 spectrometer in attenuated total reflectance (ATR) mode. X-ray photoelectron spectroscopy (XPS) measurements were carried out on an ESCALAB 250 Xi system employing monochromatic Al/K_*α*_ excitation. The in situ XRD test, in situ Raman test, and ex situ characterizations can see more discussion in supporting information.

### Electrochemical Measurements

HC electrodes were fabricated in the following steps: (1) mix 80 wt% as-prepared HCs, 10 wt% Super P conductive carbon, and 10 wt% PVDF binder in NMP to form a homogeneous slurry. (2) The slurry was uniformly coated onto copper foil and vacuum-dried at 60 °C for 10 h, yielding an active material loading of ~ 1.0 mg cm^−2^. (3) The as-dried electrodes were cold rolled at a rolling pressure of 10 ton. The electrochemical performance of the as-prepared HC samples was then evaluated using CR2032 coin cells assembled in an argon-filled glove box. The half-cells were constructed with sodium metal disks serving as counter/reference electrodes and HCs as the working electrode. For coin-type full-cell assembly, a half-cell was first constructed using an HC-1300 anode (derived from B-stage resin carbonized at 1300 °C) and Na metal. The half-cell was cycled for five cycles at 0.1 A g^−1^ to a charged state of 2.5 V, after which it was disassembled and the pre-cycled HC-1300 electrode was collected. The resulting pre-cycled HC-1300 anode was then paired with a Na_3_V_2_(PO_4_)_3_ (NVP) cathode to assemble the coin-type full cells, with the positive/negative (P/N) capacity ratio precisely controlled at 1.05. The ether-based electrolyte system comprised 1.0 M NaPF_6_ dissolved in DME (NP-035 purchased from DodoChem, 225 μL), while glass fiber separators (Whatman GF/D) were implemented to prevent electrode shorting. All galvanostatic charge–discharge cycling tests were conducted using a NEWARE CT-4008 battery tester under voltage ranges of 0.01–2.5 V (half cells) and 1.6–3.5 V (full cells). Cyclic voltammetry (CV) tests were conducted using a Bio-Logic electrochemical workstation. Galvanostatic intermittent titration technique (GITT) measurements were carried out on the same NEWARE system with identical voltage cutoffs.

In pouch cell configurations, commercial Na_4_Fe_3_(PO_4_)_2_P_2_O_7_ (NFPP), purchased from Shenzhen OME. Co., was used as the cathode and HC-1300 as the anode (a mass loading of ~ 6.3 mg cm^−2^), with a rigorously controlled positive/negative (P/N) capacity ratio of 1.2:1. Al foil was used as the current collector for the anode. The pouch cells were assembled in Ar-filled glovebox with polypropylene membrane as the separator and NP-035 as the electrolyte (3.5 mL). Galvanostatic charge–discharge cycling tests were systematically performed using a NEWARE CT-4008 battery testing system within an operational voltage range of 1.0–4.0 V.

The energy densities of the sodium-ion full cell are calculated by numerically integrating the galvanostatic discharge profiles using Eq. 1 [26]:

1 $$E = \int_{{{\mathrm{t1}}}}^{{{\mathrm{r2}}}} \frac{UI}{m} {\mathrm{d}}t$$

where *m* refers to the total mass (kg) of the anode and cathode. *I* and *U* represent the discharge current (A) and operating voltage (V), respectively. *T* (s) is *t*_1_ (start time of the discharge) − *t*_2_ (end time of the discharge).

Ex situ characterizations. Half-cells discharged to 0.01 V were disassembled in an argon-filled glovebox (O_2_/H_2_O < 0.1 ppm), and the fully sodiated electrodes were rinsed thoroughly by dimethoxyethane (DME) solvent, followed by vacuum drying. For ex situ XPS tests, Ar^+^ ion sputtering at 1 keV was used to etch the surface of HCs before XPS analysis. For ex situ high-resolution TEM (HRTEM), the active materials were collected via mechanical scraping, which was then ultrasonically dispersed in fresh DME solvent for sample preparation.

In situ Raman test. Raman spectra were acquired using a HORIBA XploRA PLUS spectrometer equipped with a 532 nm excitation laser. Electrodes were fabricated by homogenizing active materials with polyvinylidene fluoride (PVDF) binder in a 9:1 mass ratio using N-methyl-2-pyrrolidone (NMP) as solvent, followed by blade-coating onto porous copper foil current collectors. In situ electrochemical Raman characterization was performed in a customized spectro-electrochemical cell with a quartz plate, employing 120s integration time per spectral acquisition. Electrochemical testing was performed within a voltage window of 0.01–2.5 V at a constant current density of 100 mA g^−1^. All optical measurements were conducted under ambient conditions with thermal equilibrium verified prior to testing.

In situ XRD test. Work electrodes were fabricated with 90 wt% active material and 10 wt% polyvinylidene fluoride (PVDF) binder. Operando characterization was conducted using a customized in situ electrochemical cell equipped with a beryllium (Be) window for X-ray diffraction (XRD) monitoring. Electrochemical testing was performed within a voltage window of 0.01–2.5 V at a constant current density of 100 mA g^−1^.

### Computation Methods

First-principles calculations were performed by using the Vienna ab initio Simulation Program (VASP) [37, 38]. The Perdew–Burke–Ernzerhof (PBE) exchange–correlation functional [39] and the projector-augmented wave approach [40] were adopted. The van der Waals corrections was taken with the Semiempirical Grimme parameter DFT-D3 correction [41]. The convergence criteria of structure optimization were chosen to be the maximum force on each atom less than 0.01 eV/Å with an energy change less than 1 × 10^−6^ eV. Bilayer graphene sheet calculations were performed using slab configurations with a vacuum of ~ 15 Å in *c* direction, where the cut-off energy for planewave basis set and gamma-centered [42] k-point for sampling the Brillouin zones were selected to be 400 eV and 3 × 3 × 1, respectively. The calculation was conducted in two subsequent relaxation steps: first, with increased layers of pure metallic Na included in the bilayer graphene sheets, both the carbon matrix and Na were relaxed, from which the interlayer spacings of the carbon matrix were determined to be 0.82, 1.05, 1.19, 1.48, 1.82, and 2.18 nm, respectively. Next, the electrochemical process was simulated by filling the graphitic pore with Na atoms one by one in energy competing positions and performing geometrical and energy optimizations, where the carbon matrix was locked in position, allowing only Na to relax. The enthalpies of formation (*H*) were obtained based on the calculated energies according to the following formula [43–45]:

2 $$H = E_{{{\mathrm{Na}}_{x} {\text{ + C}}_{n} }} - E_{{{\mathrm{Na}}_{x} {\mathrm{C}}_{n} }} - E_{{{\mathrm{Na}}}}$$

Where $E_{{{\mathrm{Na}}_{x} {\mathrm{C}}_{n} }}$, $E_{{{\mathrm{c}}_{n} }}$ and $E_{{{\mathrm{Na}}}}$ represent the total energies of *x*Na in the carbon matrix (C_*n*_), carbon matrix, and the Na atom on the surface of its metallic crystal form, respectively. *H* < 0 corresponds to *V* > 0.

Ab initio molecular dynamics (AIMD) simulations were performed using cut-off energy of 400 eV and k-point mesh of 1 × 1 × 1. The Nosé-Hoover thermostat was used to run all the AIMD simulations in the canonical ensemble (NVT), which has a constant number of atoms, volume, and temperature [46, 47]. Newton’s equations of motion were integrated using Verlet’s logarithm in the velocity form with a time step of 1 fs at 300 K for all the structures. 18 DME molecules and 2 Na atoms were packed randomly in a vacuum space of 16.79 × 9.95 × 19.82 Å^3^ and pre-conditioned for 10 ps to simulate the electrolyte environment. The number of solvent molecules was evaluated by using the density of liquid solvent (0.87 g cm^−3^ for DME). Next, a discharged graphitic electrode was constructed on the left side of the electrolyte box. Excess Na atoms were sandwiched between two graphene nanosheets, and two sets of this sandwiched structure were separated by *d* spacings of 0.6, 0.8, and 1.0 nm, respectively. Another graphene nanosheet was positioned vertically to set a periodic boundary condition. The solid-electrolyte interface was passivated by H atoms. Finally, an electrochemical half-cell consisting of 682 atoms was constructed, with a total dimension of 16.79 × 9.95 × 42.96 Å^3^. During AIMD, the left part of the simulation cell was fixed in order to maintain the electron-enriched nano-space with pre-determined *d* spacing. The average potential of the intercalation reaction was evaluated using Eq. 3, by calculating the total energies of the optimized intercalated structure as $E_{{{\mathrm{Na}}_{x + y} {\mathrm{C}}_{n} }}$ and the optimized structure with one less Na atom before intercalation as $E_{{{\mathrm{Na}}_{x} {\mathrm{C}}_{n} }}$.

3 $$V = \frac{{ - (E_{{{\mathrm{Na}}_{x + 1} {\mathrm{C}}_{n} }} - E_{{{\mathrm{Na}}_{x} {\mathrm{C}}_{n} }} - E_{{{\mathrm{Na}}}} )}}{e}$$

For data analysis and molecular visualizations, the application VMD [48] and Materials Studio were utilized.

## Results and Discussion

### General Considerations of Nano-Space Confinement

Owing to the quasi-metallic nature, Na-cluster storage in the closed pores of HCs is expected to proceed through nucleation and growth stages, analogous to the electroplating of bulk metals on surfaces. A key distinction, however, is that these stages are further modulated by confined nano-space within the orifice and cavities of the closed graphitic pores. We begin our discussion with the theory of heterogeneous nucleation, which defines a critical nucleus size governed by the surface properties of the Na clusters, the graphitic walls, and the interactions between them (Fig. S1). Once this size is surpassed, the clusters continue to grow. The critical radius of the nucleus (r^*^) and the activation free energy (∆G^*^) of heterogenous nucleation can be described according to Eqs. 4 and 5 [49, 50]:

4 $$r^{*} = - \frac{2\gamma }{{\Delta H_{f} }} \cdot \frac{{T_{m} }}{{T_{m} - T}} = - \frac{2\gamma }{{\Delta H_{f} }} \cdot \frac{{T_{m} }}{\Delta T}$$

5 $$\Delta G^{*} = \left( {\frac{{16\pi \gamma^{3} }}{{3\Delta H_{f}^{2} }}} \right) \cdot \left( {\frac{{T_{m} }}{\Delta T}} \right)^{2} \cdot \left( {\frac{{2 - 3\cos \theta + \cos^{3} \theta }}{4}} \right)$$

Where *γ* is the surface free energy of the nucleus, ∆*H*_*f*_ is the specific latent heat, *T*_*m*_ is the solidification temperature of the nucleus, ∆*T* is the degree of supercooling, *θ* is the contact angle (Fig. S1a, b). In electrochemical deposition, *T*_*m*_ and ∆*T* can be understood as the equilibrium potential and overpotential of the solidification reaction, respectively.

**Fig. 1:**
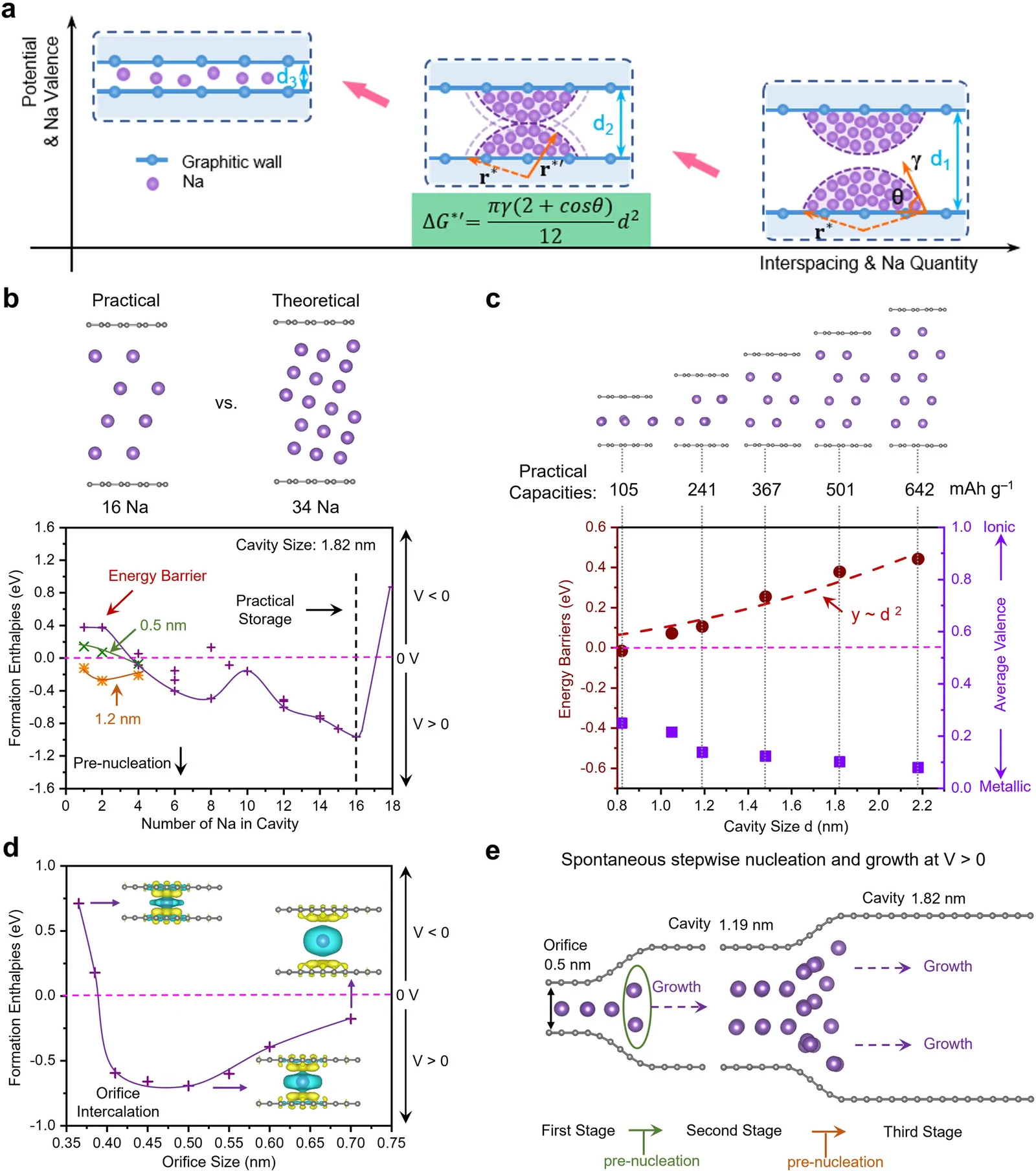
Theoretical considerations of nano-space confinement on Na storage in closed pores. **a** Heterogenous nucleation under nano-space confinement, with a new equation derived and shown in the green panel. **b** Formation enthalpy changes with the number of Na stored in a cavity of size 1.82 nm. The energy barrier can be reduced by pre-nucleation in a 0.5 nm-orifice (green line) or in a 1.2 nm-cavity (orange line). The optimized sodiation structures with (practical) and without (theoretical) considering energy compensation. **c** Variation of the energy barrier and average Bader valence with cavity size, providing practical capacities based on the optimized sodiation structures. **d** Formation enthalpy changes with orifice size. Insets are charge density difference diagrams for the orifices of 0.375 nm, 0.5 nm, and 0.7 nm. **e** Calculated pre-nucleation structures for the connected pores of 0.5 nm-orifice//1.2 nm-cavity and 1.2 nm-cavity//1.82 nm-cavity, illustrating spontaneous stepwise nucleation and growth at positive potentials. Pink: Na; gray: C; yellow: electron rich; cyan: electron deficient. Isosurface: 0.0009

As illustrated in Fig. 1a, as the size (*d*) of the closed pore decreases, two embryos touch and r^*^ decreases, then the new critical radius of the nucleus ($\mathop r\nolimits^{{ *^{\prime}}}$) can be related to $d^{\prime}$ in Eq. 6 (Fig. S1c):

6 $$r^{\prime\prime} = \frac{{d^{\prime}}}{2(1 - \cos \theta )}$$

Assuming that $\mathop r\nolimits^{{ *^{\prime}}}$ follows the condition given by Eq. 5 (Fig. S1b), by substituting Eq. 6 into Eq. 4 and Eq. 5, we can obtain the following equation (Eq. 7):

7 $$\Delta G^{*\prime } = \frac{\pi \gamma (2 + \cos \theta )}{{12}}d^{\prime 2}$$

Equations 6 and 7 suggest that as the available space becomes more confined, the critical nucleus size decreases in a square relationship, thereby favoring nucleation at lower energy compensation in a nanoconfined space. This underscores the favorable role of nano-space confinement in promoting the formation of Na clusters within the cavities of closed graphitic pores. However, the question remains whether Na clusters grow spontaneously within the decreasing size of cavities at positive potentials (*V* > 0).

To elucidate the effects of nano-space confinement on the form of Na deposited within closed pores at positive potentials (*V* > 0), DFT calculations were performed further. Firstly, we built sodiation models with 2 to 7 layers of bulk metallic Na contained within graphitic cavities. After optimization without fixation of the graphitic walls, the interlayer spacings of 0.82, 1.05, 1.19, 1.48, 1.82, and 2.18 nm were obtained. Secondly, with these interlayer spacings fixed, the electrochemical processes were then simulated by filling the corresponding the graphitic cavities with Na atoms one by one in energy competing positions and performing geometrical and energy optimizations. On the basis of the lowest enthalpy path, we can obtain formation enthalpy (*H*) profiles along with the Na sodiation process (Fig. S2). The enthalpies of formation were obtained according to previous studies [51–53], where *H* < 0 corresponds to the average potentials *V* > 0. Accordingly, formation enthalpies located below the pink dashed line in Figs. 1b and S2 indicate energetically favorable Na storage at *V* > 0.

A typical example of a 1.82 nm cavity is shown in Fig. 1b. If the stored Na maintains the crystal structure of metallic Na, theoretically, 34 Na atoms can be accommodated within the 1.82 nm cavity. Interestingly, the electrochemical process simulated in Fig. 1b shows that after storing 16 Na atoms, the formation enthalpy rises sharply to a high positive value, indicating that only 16 Na atoms can be practically stored in the confined 1.82 nm cavity at *V* > 0. This may be one of the factors contributing to the formation of quasi-metallic Na clusters rather than bulk Na metalin the closed pores. Energy barriers of nucleation are also present in Figs. 1b and S2. Based on these data (red dots in Fig. 1c), the energy barriers can be correlated with cavity size in a square relationship (red dashed line in Fig. 1c), which corresponds well with the trend predicted by Eq. 5. Moreover, the purple squares in Fig. 1c show that the average Bader valence of the stored Na clusters decreases with increasing cavity size. Therefore, combining the number of Na atoms practically stored and the corresponding average valence, the capacity can be calculated to be 501 mAh g^−1^ for 1.82 nm cavities and 642 mAh g^−1^ for 2.18 nm cavities. This result clearly demonstrates the capacity potential of Na storage in large closed pores.

Therefore, although the energy barrier for Na-cluster growth decreases as the nanocavity size decreases, it remains energetically unfavorable at *V* > 0. More importantly, both Eq. 5 and Fig. 1c highlight a trade-off: while large cavities favor high capacity, they also demand greater energy input (i.e., larger $\Delta G^{ * \prime }$ or higher energy barrier) for Na-cluster nucleation.

Fortunately, nano-space confinement plays a crucial role once again. As also shown in Fig. 1c, when the interlayer spacing is smaller than 0.82 nm, the energy barrier disappears. This prompts us to conduct further calculations on the formation enthalpies of intercalated Na–C compounds when the interlayer spacing between two graphitic walls is smaller than 0.7 nm (i.e., the orifice of closed pores). Figure 1d shows that the intercalation of Na into the orifice between 0.39 and 0.7 nm is energetically favorable at *V* > 0. This is understandable because, in such highly confined spaces, each Na atom interacts strongly with both graphitic walls, rather than only one as assumed in the derivation of Eq. 7, thereby reducing the energy cost associated with smaller exposed surface.

Motivated by this result, we constructed complete closed pores by connecting orifices with different cavities and re-simulated the electrochemical process in these cavities. One typical example is shown on the left part of Fig. 1e. It is interesting that the spontaneous Na–ion intercalation in the 0.5 nm orifice at *V* > 0 can reduce the energy barrier to negative values for Na-cluster nucleation in the cavities up to 1.48 nm (green lines in Fig. S2b–d). However, the energy barrier remains positive for the closed pore with a 0.5 nm orifice and a 1.82 nm cavity (the green line in Fig. 1b). Thus, we next constructed a two-stage cavity by linking the 1.2 nm cavity to the 1.82 nm cavity, as shown on the right side of Fig. 1e. In this case, the energy barrier decreases to negative values (the orange line in Figs. 1b and S2e). Thus, when the size difference between the orifice and cavity, and between adjacent cavities, is sufficiently small, spontaneous nucleation and growth of Na clusters occur in progressively larger cavities at positive potentials, thereby raising the deposition potential of quasi-metallic Na clusters within closed pores above zero. This clearly suggests that a highly coupled “intercalation-pore filling” and stage-wise storage process is effective for Na-cluster deposition into large cavities, enabling high capacities.

Based on our calculations in Fig. S2, the second-stage cavity should range from 0.82 to 1.48 nm, and the third-stage cavity should range from 1.48 to 2.18 nm. Having more than three stages may complicate fabrication. Therefore, we propose a stage-wise closed pore structure with an average cavity size around 2.0 nm as a rational and feasible design, potentially providing a practical capacity exceeding 500 mAh g^−1^. It should also be noted that the above discussion is simplified by neglecting various defects within and outside the closed pores. These effects will be considered in our subsequent discussion.

### Superior Na Storage in Stage-Wise Closed Pores

**Fig. 2:**
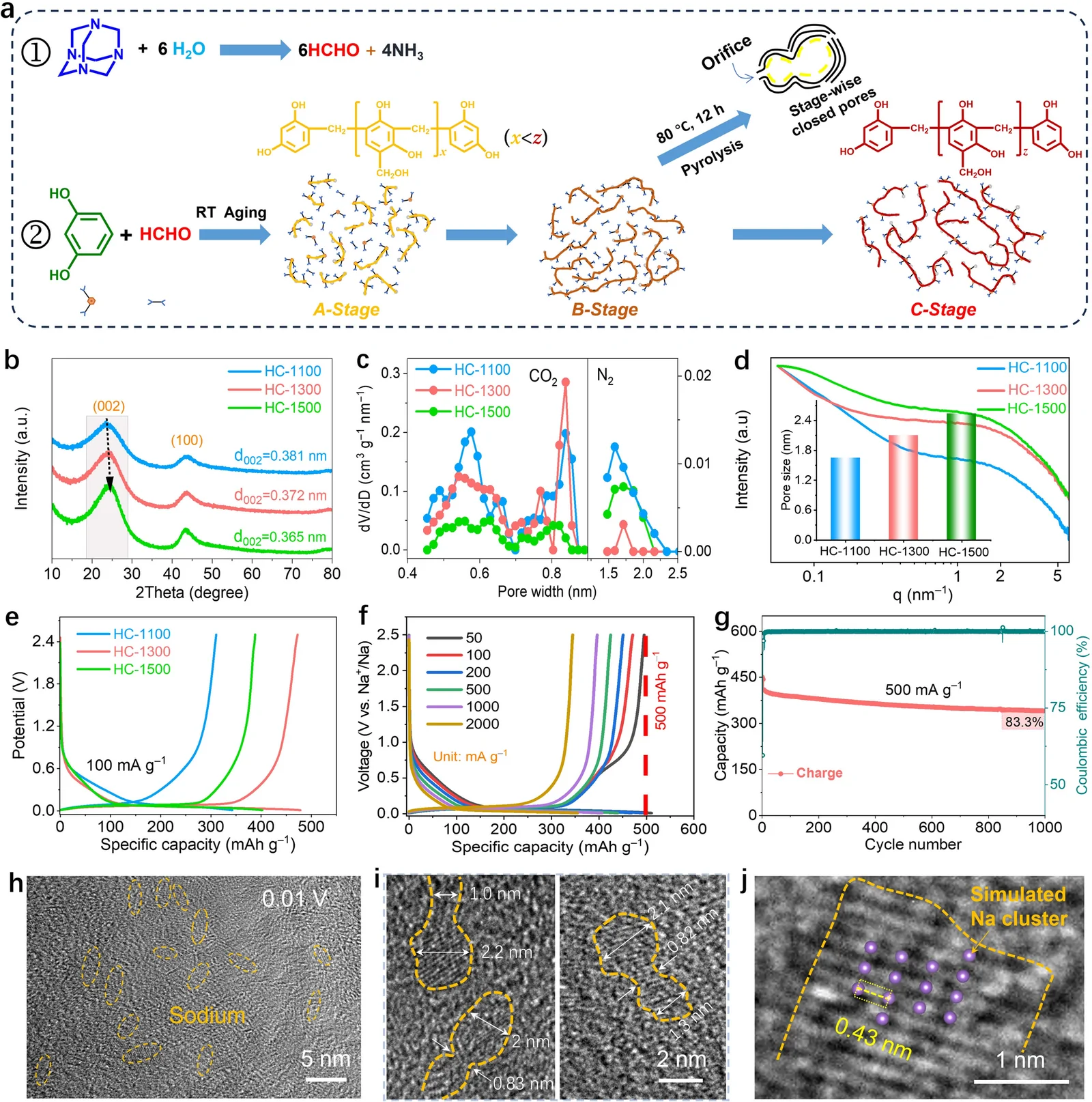
Fabrication and characterization of stage-wise closed pores. **a** Fabrication route of controlling the degree of crosslinking in the prepolymer structure of resin prior to thermo-polymerization at 80 °C. **b** XRD patterns, **c** pore size distributions, and **d** SAXS curves of different HC samples, and the inset in (**d**) is the calculated average size of closed pores. Charge–discharge profiles **e** of different HC electrodes at 100 mA g^−1^ and **f** of the HC-1300 electrode at different current densities in the third cycle. **g** Cyclic performances of the HC-1300 electrode at 500 mA g^−1^. **h-j** Ex situ HRTEM images of Na clusters in HC-1300 after fully discharged to 0.01 V, and the inset in (**j**) is the optimized structure of the Na cluster practically stored in a 1.82 nm cavity

To fabricate stage-wise closed pores, resin, as a potential precursor for commercial HCs [54–56], was chosen as the precursor. Apart from previous studies of resin-based HCs [57, 58], we sought to control the degree of crosslinking in the prepolymer structure of resin prior to thermo-polymerization. In this work, resorcinol (R) and hexamethylenetetramine (HMTA) were mixed in ethanol–water solvent (7:2 v/v). As shown in Fig. 2a, HMTA first undergoes hydrolysis to produce formaldehyde (HCHO) and ammonia (NH_3_), where NH_3_ continue to serves as a weak basic catalyst to regulate the polycondensation reaction rate between HCHO and R [59]. The HCHO to R ratio is controlled to be 1.8:1. After aging for 12 h (A-Stage), 48 h (B-Stage), and 120 h (C-stage) at room temperature, polycondensation reaction progresses but no gelation forms (Figs. 2a and S3), implying the suppressed crosslinking during aging. Subsequently, the aged solutions were thermo-polymerized at 80 °C for 12 h to initiate substantial crosslinking, resulting in powders from the A-Stage and large resin chunks from the B-Stage and C-Stage. After grinding, the resin powders were subjected to progressive pyrolysis at elevated temperatures. Based on the performance shown in Figs. S4 and 2, B-Stage resin was finally selected for the fabrication of HCs with the targeted pore structure and for detailed analysis.

The evolution of the graphitic and pore structures in B-Stage resin-derived HCs has then been investigated with increasing pyrolysis temperatures from 1100 to 1500 °C. The as-prepared HC powders are denoted as HC-1100, HC-1300, and HC-1500. X-ray diffraction (XRD) patterns in Fig. 2b show a gradual shift of the (002) diffraction reflexes to higher angles as the pyrolysis temperature increases, corresponding to a reduction in the *d*-spacing of the graphitic domains from 0.386 to 0.365 nm. Scherrer analysis reveals the dimensional growth of the graphitic domains, as evidenced by the increase in *L*_*a*_ and *L*_*c*_ values (Table 1 and Fig. S5) [60, 61], confirming the temperature-assisted graphitization process. Ex situ Raman spectroscopy (Fig. S6) indicates a decrease in structural defects, with the *A*_*D*_/*A*_*G*_ ratio reducing from 2.28 to 1.26 [62]. All these results confirm that as the pyrolysis temperature increases, the graphitic interlayer spacing shrinks, the graphitic domains grow, and the graphitic defects are reduced.

**Table 1:** Physical parameters of HC-1100, HC-1300, and HC-1500 samples

Sample	*d* [Å]	*L*_*a*_[nm]	*L*_*c*_[nm]	*S*_BET_ [m^2^ g^−1^]	*A*_*D*_/*A*_*G*_	Open pore volume [cm^3^ g^−1^]	True density [g cm^−3^]	Closed pore volume [cm^3^ g^−1^]	Average closed pore size[nm]
HC-1100	3.81	10.61	0.92	76.30	1.51	0.034	2.190	0.014	1.6
HC-1300	3.72	11.45	1.02	66.18	1.40	0.027	1.900	0.084	2.0
HC-1500	3.65	12.71	1.06	45.63	1.26	0.021	2.032	0.050	2.5

Gas physisorption analyses, small-angle X-ray scattering (SAXS), and true density measurements were carried out to investigate temperature-dependent evolution of pore structures. Open pores can be detected by gas physisorption analyses using N_2_ (77 K) and CO_2_ (273 K) probes (Fig. S7a). The pore size distribution combining CO_2_ and N_2_ data (Fig. 2c) show three characteristic ranges: 0.45–0.65, 0.65–0.85, and 1.5–2.3 nm. Quantitative analysis of open pore volume is present in Fig. S7b and summarized in Table 1. It is shown that, with increasing pyrolysis temperature, small open micropores coalesce to larger ones, while some open micropores collapse to form new surfaces, resulting in a reduction of the total open pore volume at higher temperatures. The observed trend for open pores is expected to apply to closed pores as well. SAXS patterns of HCs show a broad peak around *q* = 0.5 nm^−1^, indicating a distribution of closed nanopores (Fig. 2d) [29]. Based on the spherical closed pore model, the characteristic length related to the variation in scattering intensities was fitted (Fig. S8) [56], providing the average diameters of the closed pores increasing from 1.6 nm for HC-1100 to 2.0 nm for HC-1300, and further to 2.5 nm for HC-1500. In the true density (*ρ*_true_) test, helium gas was used to obtain the density of carbon skeleton, as helium can enter all open pores at room temperature [63, 64]. The closed pore volume was then calculated using the formula $V_{{\text{closed pores}}} = 1/\rho_{{{\mathrm{true}}}} - 1/2.26$ [65]. Table 1 reveals a sharp increase in the closed pore volume from 0.014 cm^3^ g^−1^ for HC-1100 to 0.084 cm^3^ g^−1^ for HC-1300. This result implies the significant formation of small closed pores and the onset of pore coalescence at 1300 °C, which is beneficial for the development of stage-wise closed pores. The closed pore volume decreases from HC-1300 to HC-1500 (0.050 cm^3^ g^−1^), indicating the general coalescence of small pores into larger ones, with some collapsing into open pores (Table 1). Considering the pore structure evolution process, HC-1300 is expected to possess stage-wise closed pores with an average size around 2.0 nm, optimal for superior Na storage as predicted in Fig. 1.

For experimental validation, the Na-storage performance of the three HC electrodes was evaluated using a half-cell configuration with an electrolyte of 1 M NaPF_6_ dissolved in 1,2-dimethoxyethane (DME). As expected, the HC-1300 electrode demonstrates the best performance among all three electrodes (Figs. 2e and S9). Specifically, the HC-1300 electrode can deliver a high capacity of 500 mAh g^−1^ at 50 mA g^−1^, a high-rate capability of 344 mAh g^−1^ at 2000 mA g^−1^, and 83.3% capacity retention over 1000 cycles at 500 mA g^−1^ (Fig. 2f, g). More importantly, it also exhibits a high reversible capacity of 388.5 mAh g^−1^ at 100 mA g^−1^ even at a high areal loading of 3.7 mg cm^−2^ (Fig. S10). To visualize the stage-wise closed pores, a reliable approach is to allow Na clusters to inflate the closed pores and then characterize their shape and structure using high-resolution transmission electron microscopy (HRTEM). Accordingly, the HC-1300 electrode was discharged to a fully sodiation sate at 0.01 V and examined by ex situ HRTEM. Figures 2h, i and S11 demonstrate that, after being inflated by Na clusters, the closed pores adopt a stage-wise geometry, with the second-stage cavity size ~ 1.0 nm and the third-stage cavity size ~ 2.0 nm. Although the orifice is difficult to clarify in this approach of characterization, its presence is evident in Figs. S12and S13 before sodiation, as Na atoms need to pass through the orifice for the formation of Na clusters in the closed pores. Meanwhile, Fig. S14 demonstrates that Na is uniformly distributed in HC-1300. More importantly, the embedded Na cluster exhibits a highly defective crystal structure in Fig. 2j. Using the optimized crystal structure of the practically stored Na cluster in the 1.82 nm cavity (Fig. S15b) to fit the main matrix in Fig. 2j yields a consistent match, confirming the validity of our calculations in Fig. 1b. For comparison, HRTEM images of the fully discharged HC-1100 and HC-1500 electrodes are also presented. As shown in Fig. S16, only a few small, stage-wise Na clusters are present in HC-1100, whereas Fig. S17 reveals large closed pores that remain partially unfilled. Neither represents the most favorable pore size or geometry for achieving high capacities, as predicted theoretically in Figs. 1e and S2. Moreover, the HRTEM images obtained after 50 cycles (Fig. S18) reveal that the closed-pore structure in HC-1300 remains intact, with no obvious collapse under the mechanical stresses induced by the repeated deposition and dissolution of Na clusters. These findings further confirm that the structural stability of closed pores in HCs is essential for their long-term cycling stability during Na storage.

### Nano-Space Confinement for Plateau Region

**Fig. 3:**
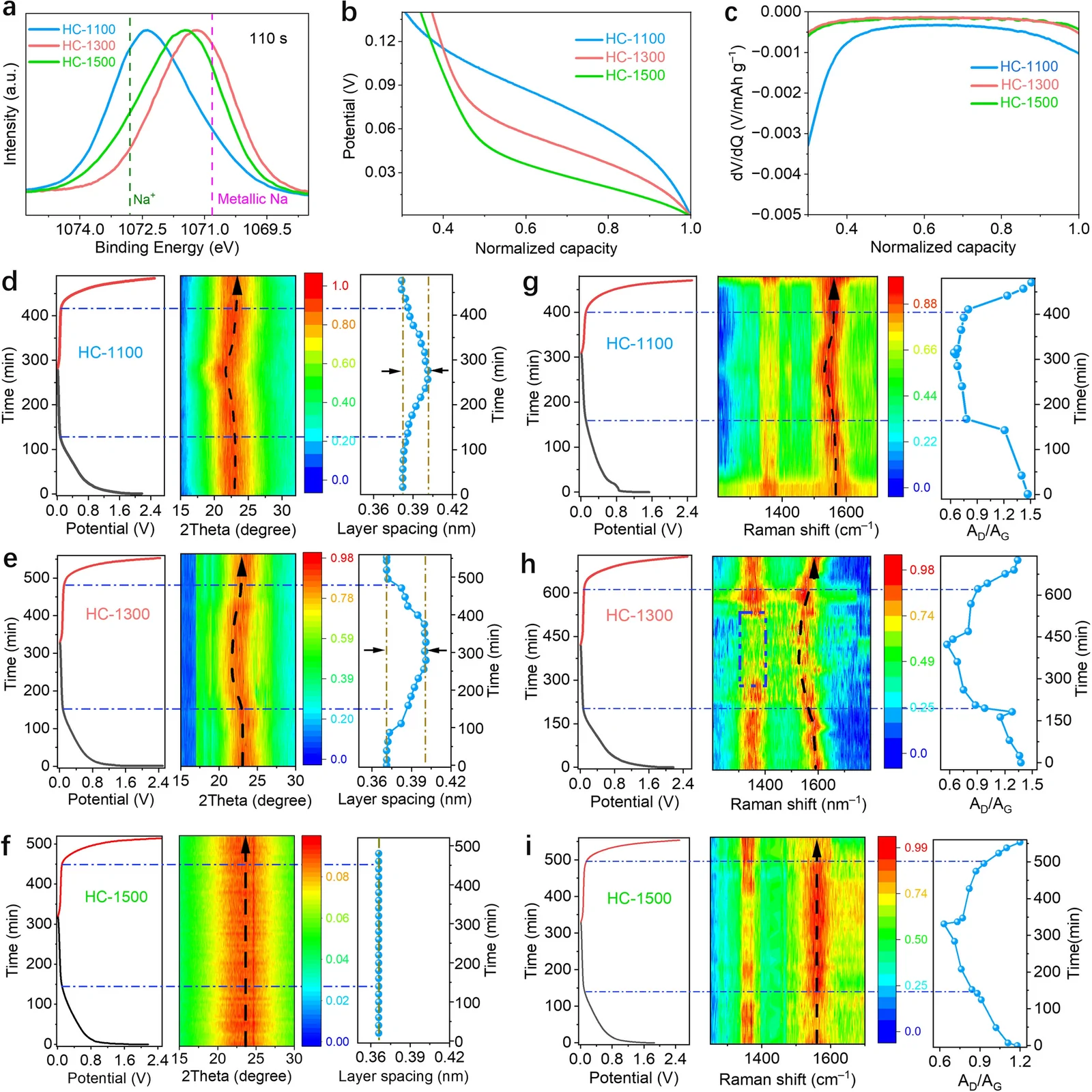
Understanding nano-space confinement for plateau region. **a** XPS spectra of Na 1s spectra of HC-1100, HC-1300, and HC-1500 at the full sodiation state (0.01 V) after Argon etching of 110s. **b** Discharging potential versus normalized discharge capacity for three HC electrodes. **c** The corresponding differential curves of (**b**). **d–f** The operando XRD patterns and **g–i** the operando Raman mappings of three HC electrodes during the first charging-discharging cycle

The systematic variation in the pore structures of HC-1100, HC-1300, and HC-1500 provides an excellent materials platform for revisiting the Na-storage mechanism by integrating the effects of nano-space confinement. Since pore filling occurs in the plateau region, the state of Na clusters stored in the stage-wise closed pores were examined first, by the reaction between the fully sodiated HC electrodes and ethanol (Fig. S19 and Movies S1–S3). The ion conductivity test (Fig. S19b), FTIR, and ^1^H NMR (Fig. S20) of the resulted solution all suggest the quasi-metallic state of Na clusters formed in the plateau region. Furthermore, X-ray photoelectron spectroscopy (XPS) analysis was carried out (Figs. 3a and S21), after 110s of sputtering to remove the solid-electrolyte interface (SEI) film from the surface of the fully sodiated HC electrodes. Figure 3a shows that the binding energies of Na clusters in all three samples fall between those of metallic Na^0^ and ionic Na^+^, confirming the co-existence of intercalated Na ions and quasi-metallic Na clusters in the fully sodiated HCs. Notably, the state of Na stored in HC-1100 appear to be more ionic than those in HC-1300 and HC-1500, suggesting that the dominant state of Na in the fully sodiated HC-1100 is the intercalated Na ions.

To fully explore the Na-storage mechanism in the plateau region, the enlarged low-potential plateau regions of the discharge profiles are shown in Fig. 3b, with the corresponding differential potential vs. normalized capacity profiles displayed in Fig. 3c. These results are analyzed in conjunction with the in situ XRD and Raman data presented in Fig. 3d–i. The apparent potential inflection point in the middle of the discharge curve of the HC-1100 electrode indicates that the dominant Na-storage mechanism in HC-1100 is intercalation [66], consistent with the XPS results in Fig. 3a. This is further confirmed by the in situ XRD diagram of the HC-1100 electrode in Fig. 3d, which shows a downshift of the (002) reflex, indicating an expansion of *d* spacing in the plateau region. In contrast, for the HC-1500 electrode, the plateau region appears as an inclined line, with no inflection point present throughout the discharge curve (Fig. 3b, c). This clearly indicates that the dominant Na-storage mechanism in HC-1500 is pore filling [66]. This is also aligned with in situ XRD diagram of the HC-1500 electrode in Fig. 3f, where no shift of the (002) reflex is shown. Up to this point, the Na-storage behavior can be understood by the “adsorption-intercalation/filling” mechanism [22, 67].

Here comes new insights. The plateau potential for the HC-1100 electrode is higher than that for the HC-1500 electrode. According to Fig. 1d, Na-ion intercalation into the graphitic walls with a *d*-spacing smaller than 0.7 nm is energetically favorable at *V* > 0, thereby occurring at higher positive potentials compared to pore filling. This potential difference explains the reason why the potential inflection point can be used as an electrochemical pointer to distinguish the dominant process. More importantly, the plateau potential for the best performing HC-1300 electrode lies between those of the HC-1100 and HC-1500 electrodes, exhibiting a continuous inclined line with the potential inflection point occurring near the end of the discharging curve. It is interesting for the HC-1300 electrode that, a downshift of the (002) reflex in the plateau region is present in the in situ XRD diagram (Fig. 3e), indicating the significant role of intercalation in HC-1300; meanwhile, the long inclined line in Fig. 3b also highlights the importance of pore filling in HC-1300. These results suggest a favorable coupled storage mechanism, where Na-ion intercalation into the orifices and Na-cluster formation in the cavities (pore filling) must be closely coupled to achieve superior Na storage. This provides clear experimental evidence to support our calculations in Fig. 1e. Specifically, an energetically favorable orifice intercalation is followed by stepwise pre-nucleation, leading to Na-cluster formation in progressively larger cavities at *V* > 0. Dominance of either process alone would diminish the overall performance.

Another important aspect can also be observed in the in situ Raman results. For the HC-1300 electrode in Fig. 3h, the D band (~ 1350 cm^−1^) almost disappears in the plateau region at the end of sodiation process (indicated by the blue frame), which reappears during the subsequent desodiation process. The weakening of the D-band in the plateau region can be ascribed to the suppression of the breathing vibration of carbon rings caused by the adsorption of Na ions at the defective sites [57]. Because the intercalation and pore filling are highly coupled for the HC-1300 electrode, the disappearance of the D band in the plateau region in Fig. 3h suggests the adsorption of Na ions at the defective sites within the cavities. Therefore, in addition to orifice-assisted and stepwise nucleation, these internal defects can also serve as additional energy favorable nucleation sites for Na-cluster formation (Fig. S22), thereby reducing the energy barrier and facilitating pore filling in larger cavities. Using the disappearance of the D band in the plateau region as the starting point, the contribution of internal defect-assisted nucleation to the total capacity was estimated to be 27.7% of the total capacity. This might open up new pathways for further enhancing Na-storage performance. A similar change in the D band can be observed for the HC-1100 electrode (Fig. 3g), but it is much less pronounced for the HC-1500 electrode (Fig. 3i) due to the reduced graphitic defects at the high pyrolysis temperature of 1500 °C (Fig. S6).

### Nano-Space Confinement for Slope Region

Before addressing the nano-space confinement effects in the slope region, we have to clarify the orifice size of closed pores in HCs, which plays a key role for Na storage in the closed pores as discussed in Fig. 1. In the true density (*ρ*_true_) test, helium gas is considered to enter all open pores at room temperature [29, 68]. Thus, any orifice that helium atoms cannot penetrate should be regarded as the orifice of closed pores. The size of a helium atom is approximately 0.26 nm [69]. Considering the π-π stacking interaction range of 0.34 nm [70], the largest *d* value for the orifice of closed pores should be at 0.6 nm. This means that, in the plateau region, we only focus on the closed pores with the orifice at ~ 0.4 nm, as obtained from the change of (002) reflex in XRD patterns (Figs. 2b and 3d–f). The significance of the closed pores with orifices between 0.4 and 0.6 nm, however, should not be neglected.

**Fig. 4:**
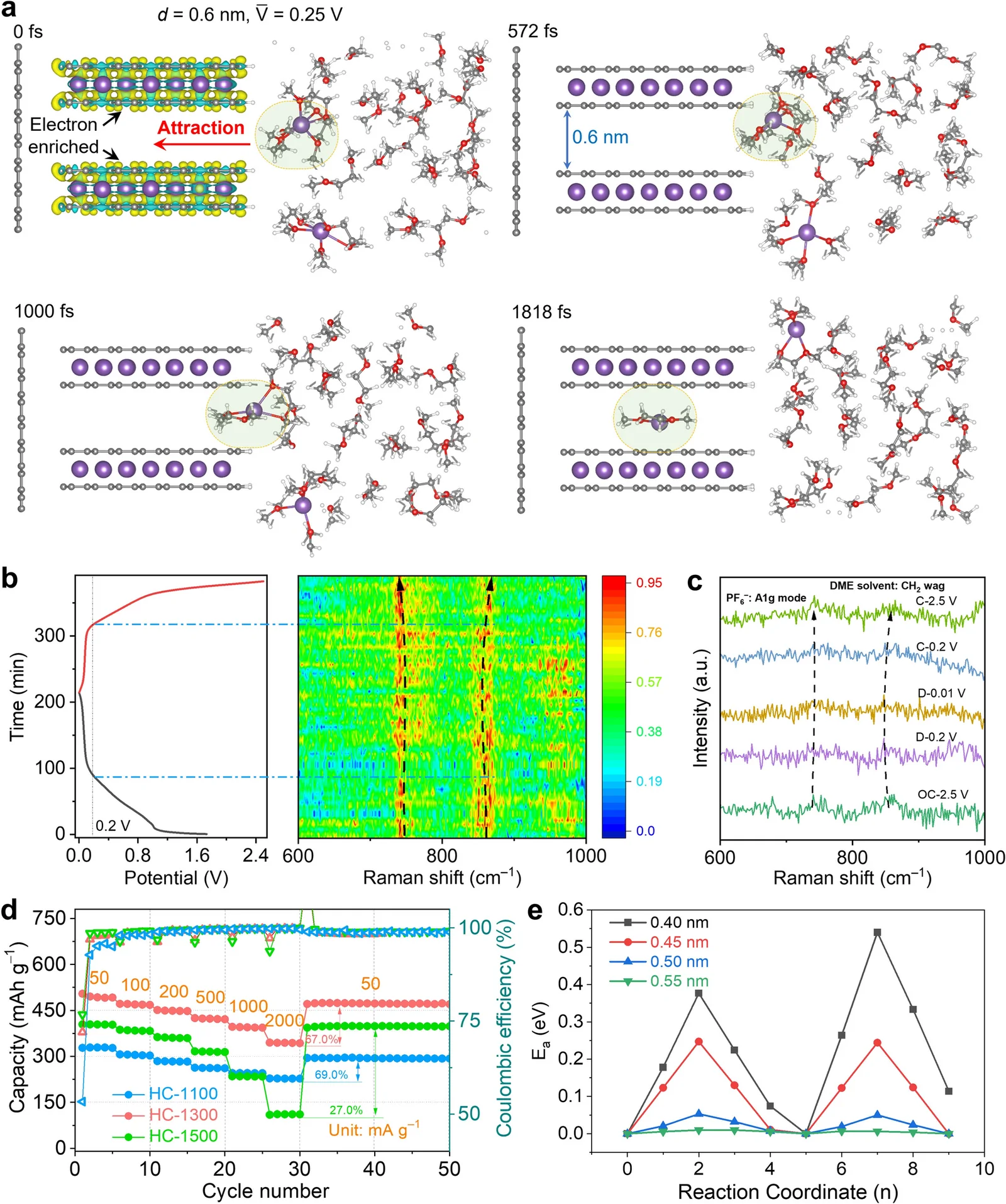
Understanding nano-space confinement for slope region and rate capability. **a** Snapshots of AIMD simulations at 0, 572, 1000, and 1818 fs revealing pre-desolvation process of Na ions into the graphitic layers with the *d*-spacing at 0.6 nm. Excess Na atoms were used to create electron-enriched internal interfaces to attract the solvated cation. Pink: Na; gray: C; white: H; red: O; yellow: electron rich; cyan: electron deficient. Isosurface: 0.003. **b** In situ Raman spectra between 600 and 1000 cm^−1^ with the corresponding charging-discharging curve, and **c** the selected Raman spectra indicating the shift of CH_2_ wagging mode. OC: open circuit. D: discharging. C: charging. **d** Rate capability tests for three HC electrodes at current densities increasing from 50 to 2000 mA g^−1^. **e** Migration energy barriers of Na-ion diffusion within the highly-confined space with the *d*-spacing ranging from 0.40 to 0.55 nm

Previous studies have shown that within relatively large interspacings, Na ions and ether-based solvent molecules can co-intercalate into the graphite layers, such as in ternary graphite intercalation compounds [71] or in the grain boundary cavities and mesopores of the microcrystalline graphite fiber [72]. Inspired by this, we investigate the effect of nano-space confinement on the pre-desolvation process. Ab initio molecular dynamics (AIMD) calculations were carried out to understand the intercalation process of Na ions into the graphitic layers with the *d*-spacing at 0.6 nm. The challenge is how to apply the appropriate negative electric field to attract solvated cations into the graphitic layers in DFT calculations. Here, we use excess Na atoms to donate electrons to the graphene layers, simulating the negative electric field applied to the anode (Fig. 4a). The structural model, although fictitious, creates electron-enriched internal interfaces within the nanoconfined space of the graphitic layers, which initiates the attraction of solvated cations. The exact electric field strength cannot be determined using this model, but fortunately, the average potential of the intercalation process can be derived from Eq. 3 in Experimental section.

The stable solvation structure of Na ions with DME molecules was first calculated using AIMD. It is shown in Fig. S23 that three DME molecules tightly surround the central Na ion, forming six Na∙∙∙O in an octahedral coordination. Movie S4 shows the intercalation process of Na ions into the graphitic layers with the *d*-spacing of 0.6 nm. Several key snapshots were captured and are present in Fig. 4a. At 572 fs, one of the surrounding DME molecules enters the nanoconfined space, dragging the Na ion along with it. At 1000 fs, the central Na ion enters the nanoconfined space, but the other two surrounding DME molecules are blocked due to steric hindrance, as they are aligned perpendicular to the graphitic layers. At 1818 fs, the entering DME molecule and Na ion form a planar geometry, facilitating their co-intercalation into the inner part of the nanoconfined space. The average potential determined for this co-intercalation process is 0.25 V, suggesting the occurrence of pre-desolvation near the end of the slope region. To verify the pre-desolvation process experimentally, we analyzed the solvation structure evolution in HC-1300 by in situ Raman spectroscopy (Fig. 4b, c). It is reported that the CH_2_ wagging mode of ether-based solvents shifts to lower wavenumbers with increasing salt concentration [73]. This aligns well with our observation in Fig. 4c, where a distinct red shift is evident after full discharge to 0.01 V, attributable to Na-ion desolvation that increases the Na-ion concentration within the closed pores of HCs.

Interestingly, in the absence of Na ions in the electrolyte, no intercalation of DME molecules occurs at *d* = 0.6 nm (Fig. S24). It shows that, without the formation of the planar geometry with the Na ion, the free-style DME molecules are still too large to be intercalated into this nanoconfined space. This is an important feature, as it differentiates ion insertion from electrolyte infiltration. The pre-desolvation near the end of the slope region, induced by nano-space confinement, is crucial for high-rate capability and subsequent pore filling, because (1) desolvation can be the rate-determining step in Na storage, and (2) co-intercalated DEM molecules influence Na-cluster nucleation within the cavities. It is noted that the presence of DME molecules alongside Na ions may be another factor contributing to the formation of quasi-metallic Na clusters rather than bulk Na metal in the closed pores. In addition, such co-intercalated solvent molecules may also affect the long-term cycling stability and Coulombic efficiency of HC anodes through SEI formation within the cavities. The discussion of nano-space confinement on the rate performance will be discussed in the next section.

For completeness, AIMD simulations were also performed on open pores with orifices larger than 0.6 nm (*d*-spacing). At a *d*-spacing of 0.8 nm, pre-desolvation of Na ions occurs (Fig. S25 and Movie S5), accompanied by the intercalation of individual DME molecules into the nanoconfined space (Fig. S26). At a *d*-spacing of 1.0 nm, the solvated Na ions undergo structural deformation rather than pre-desolvation (Fig. S27 and Movie S6), and individual DME molecules can also intercalate in this case (Fig. S28). These features align well with the mechanism of intercalation pseudo-capacitance at the middle potentials of the slope region [74]. For pores with a *d*-spacing larger than the size of the solvated Na ion, ion adsorption on the surface or defective structures should account for the capacity at the beginning of the slope region. Moreover, the discussion of the electrochemical performance of HC-1300 in ester-based electrolytes, along with its correlation with the proposed mechanism, has also been included in Fig. S29.

### Nano-Space Confinement for Rate Capability

By understanding the effects of nano-space confinement in both the plateau and slope regions, its impact on rate capability can be further explored. As the current density increase from 50 to 2000 mA g^−1^, the HC-1300 electrode exhibits a capacity retention of 68.3% at a high rate of 2000 mA g^−1^ (Fig. 4d). Although the HC-1100 electrode exhibits the lowest specific capacity at low current densities (50–500 mA g^−1^), it demonstrates a capacity retention of 69.6% under 2000 mA g^−1^, which is comparable to that of the HC-1300 electrode. In contrast, the HC-1500 electrode shows moderate capacities at current densities of 50–500 mA g^−1^ but suffers significant capacity degradation at high rates, retaining only 27.4% under 2000 mA g^−1^. To illustrate the reaction kinetics further, cyclic voltammetry (CV) tests (Fig. S30) were performed at different scan rates, ranging from 0.1 to 0.9 mV s^−1^. Two pairs of redox peaks are present, one at ~ 0.05 V and the other at ~ 0.5 V. The kinetics for each redox pair can be evaluated using the equation *I *= av^*b*^, where is *i* the peak current and the constant *b* reflects the kinetics [75–77]. A larger *b* value indicates faster reaction kinetics. When the *b* value approaches 1, it suggests a surface-controlled reaction, indicating pseudo-capacitive behavior. Conversely, when the *b* value is close to 0.5, it indicates a diffusion-controlled reaction with slow kinetics. It shows that the *b* value reaches 0.98 for the redox pair at 0.5 V (Fig. S24d), providing experimental evidence of intercalation pseudo-capacitance at the middle potentials of the slope region. Regarding the redox pair at 0.05 V, the *b* value remains high for the HC-1100 (0.74) and HC-1300(0.72) electrodes, while for the HC-1500 electrode, it drops to 0.52. This trend is also supported by the galvanostatic intermittent titration technique (GITT) tests and EIS data (Figs. S31 and S32) [78–80]. All these results indicate that the kinetics of Na ion transport and Na-cluster formation in the closed pores is faster in HC-1100 and HC-1300 than that in HC-1500.

Na-ion desolvation, Na-ion diffusion through the orifice, and Na-cluster nucleation within the closed pores are three consecutive steps that determine the kinetics of Na storage. As shown in Fig. 4a, pre-desolvation is facilitated by the orifice with the *d*-spacing of 0.6 nm. In addition, we also calculated the energy barrier of Na-ion diffusion within the highly-confined space with the *d*-spacing ranging from 0.40 to 0.55 nm (Fig. 4e). It shows that the energy barrier is negligible for orifices with *d*-spacing of 0.55 and 0.50 nm, but increases sharply to 0.25 eV for a 0.45 nm-orifice and further to 0.55 eV for a 0.40 nm-orifice. These results suggest that for closed pores, larger orifices are preferred for achieving higher kinetics, highlighting the significance of closed pores with orifices between 0.4 and 0.6 nm. Based on this, the change of the kinetics in Fig. 4d can be understood. With increasing pyrolysis temperature, the size of the orifice decreases (Fig. 2b), which reduces the kinetics of Na-ion pre-desolvation and diffusion through the orifice. Additionally, as the pyrolysis temperature increases, the size of the cavity increases (Fig. 2d) and the graphitic defects are reduced (Fig. S6), which raises the energy barrier for Na-cluster nucleation within the cavities. All these structural features contribute to the significantly reduced kinetics for Na storage in the HC-1500 electrode. Thus, benefiting from the stage-wise closed pores with the optimized orifice and cavity sizes, the HC-1300 electrode demonstrates the best combination of capacity and rate capability in Fig. 4d.

### A Unified Mechanism and Full-Cell Performance

**Fig. 5:**
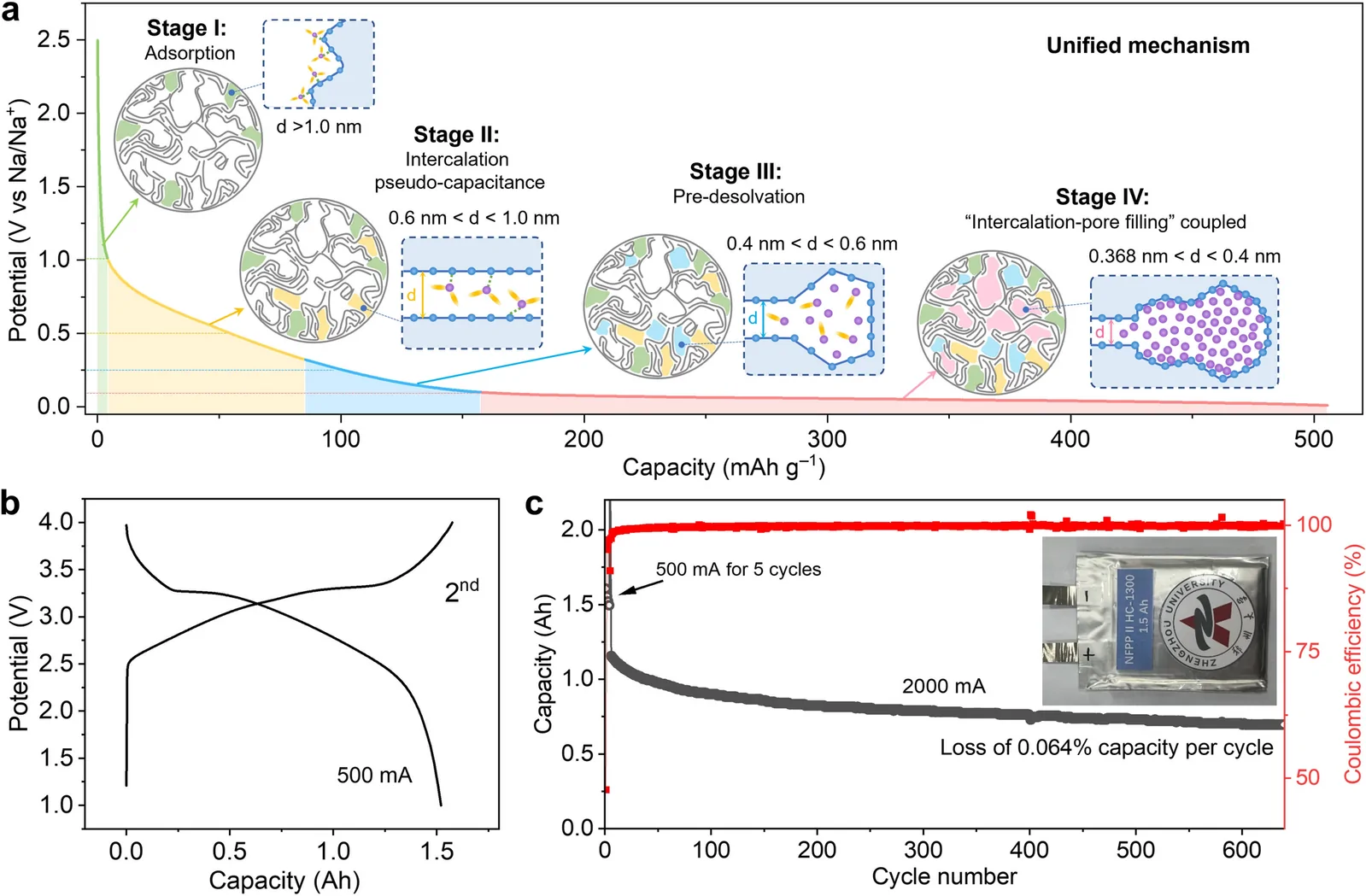
A unified mechanism and full-cell performance. **a** A unified mechanism proposed by integrating nano-space confinement effects on Na-storage behavior. **b** 1.5 Ah Na-ion pouch cells performance with charge–discharge curves at 500 mA and **c** cyclability test at 2000 mA, and the inset is a photograph of the pouch cell

By integrating nano-space confinement effects on Na-storage behavior, a unified mechanism is proposed in Fig. 5a: (1) At the beginning of the slope region, solvated Na ions adsorb on the surface, functional groups, defects, and edges of open pores (*d* > 1.0 nm) via the adsorption mechanism. (2) At the middle potentials of the slope region (~ 0.5 V), intercalation pseudo-capacitance occurs in the open pores (0.6 nm < *d* < 1.0 nm), where the solvated Na ions are deformed or partially desolvated into the open pores, accompanied by the intercalation of individual solvent molecules. After this stage, the adsorption mechanism resumes with newly accessed surfaces and defective structures. (3) Near the end of the slope region (~ 0.25 V), pre-desolvation occurs, and no individual solvent molecules can be intercalated into the orifices (0.4 nm < *d* < 0.6 nm) of the closed pores. This stage is crucial for the next-stage pore filling and high-rate capability. (4) In the plateau region (< 0.1 V), Na-ion intercalation into the orifices (0.368 nm < *d* < 0.4 nm) of the closed pores and/or pore filling in the nano-size cavities occur. Na-ion intercalation initiates stepwise pre-nucleation for the growth of Na clusters in larger cavities, while the defective structures within the cavities further facilitate Na-cluster nucleation at *V* > 0. Therefore, although either intercalation or pore filling may dominate at this stage, the highly coupled processes of intercalation and pore filling are preferred for achieving high-capacity Na storage. This unified mechanism highlights the significance of nano-space confinement, which drives the rational design of stage-wise closed pores in HCs, enabling superior overall performance of Na storage.

To demonstrate the potential of practical applications of HC-1300, coin-type full cells were assembled with Na_3_V_2_(PO_4_)_3_ (NVP) as the cathode and HC-1300 as the anode (Fig. S33a). The positive to negative ratio was controlled to be 1.2. The fabricated coin-type full cells exhibit a high initial Coulombic efficiency (ICE) of 82.6% and an average voltage of 3.25 V (Fig. S33b, c). Surprisingly, the specific capacity of the full cell maintains as high as 447 mAh g_HC_^−1^ at a current density of 50 mA g_HC_^−1^ in the second cycle. In addition, a high capacity of 306 mAh g_HC_^−1^ at 2000 mA g_HC_^−1^ and a high capacity retention of 83.9% after 200 cycles at 200 mAh g_HC_^−1^ have also been achieved in the full cell configuration (Fig. S33d). Furthermore, 1.5 Ah Na-ion pouch cells (147.4 Wh kg^−1^, Tables S1 and S2) were also assembled with commercial Na_4_Fe_3_(PO_4_)_2_P_2_O_7_ (NFPP) as the cathode and HC-1300 as the anode (Fig. 5b, c). These SIB pouch cells, with a photograph shown in the inset of Fig. 5c, exhibit only a 0.064% capacity loss per cycle over 700 cycles at 2000 mA, demonstrating the potential of using HC-1300 in real-world SIBs. While further efforts in precursor optimization and process control are still needed for large-scale implementation, the present strategy provides a reliable and promising route toward the industrial production of high-grade HCs for high-performance Na-ion batteries.

## Conclusions

To conclude, this work identifies nano-space confinement as the fundamental factor governing Na-storage behavior and establishes a unified Na-storage mechanism in HCs. A type of stage-wise closed graphitic pores with an optimal cavity size of ~ 2.0 nm was proposed, and realized experimentally through the modulation of the prepolymer structure of resins prior to pyrolysis. Superior overall performance has been achieved for HC-1300 in half-cell and full-cell configurations. Mechanistic studies show that confined nano-spaces modulate heterogeneous nucleation of quasi-metallic Na clusters within closed graphitic pores, suggesting a favorable coupled “intercalation-pore filling” and stage-wise storage mechanism. Specifically, an energetically favorable orifice intercalation is followed by stepwise pre-nucleation, leading to Na-cluster formation (pore filling) in progressively larger cavities at *V* > 0. Dominance of either Na-ion intercalation or pore filling alone would diminish the overall performance. Moreover, internal defects within the cavities can also serve as additional energy favorable nucleation sites to facilitate pore filling in large cavities. Furthermore, the importance of closed pores with orifices between 0.4 and 0.6 nm has been highlighted, which not only facilitates pre-desolvation but also enhances Na-ion transport kinetics. Thus, a new pre-desolvation stage near the end of the slope region has been clarified and incorporated into the unified mechanism. This work enriches our fundamental understanding in the correlation between the pore structure of HCs and Na-storage behavior, and may boost the overall performance of HCs beyond previous expectations.

## Supplementary Information

Below is the link to the electronic supplementary material.

Supplementary file1 (MP4 2833 KB)

Supplementary file2 (MP4 6299 KB)

Supplementary file3 (MP4 3232 KB)

Supplementary file4 (MP4 3513 KB)

Supplementary file5 (MP4 3069 KB)

Supplementary file6 (MP4 2655 KB)

Supplementary file7 (DOCX 13371 KB)

## References

[1] J. Zhang, Y. Yan, X. Wang, Y. Cui, Z. Zhang et al., Bridging multiscale interfaces for developing ionically conductive high-voltage iron sulfate-containing sodium-based battery positive electrodes. Nat. Commun. **14**(1), 3701 (2023). 10.1038/s41467-023-39384-7 37349302 10.1038/s41467-023-39384-7 PMC10287750

[2] Z. Wang, M. Ran, K. Cui, T. Li, J. Zou et al., Reinventing phosphorus anodes: taming pulverization via strain-induced interfacial coupling. Angew. Chem. Int. Ed. **65**(9), e23513 (2026). 10.1002/anie.202523513 10.1002/anie.202523513 41549935

[3] Z. Cheng, H. Zhang, J. Cui, J. Zhao, S. Dai et al., Interlayer-expanded carbon anodes with exceptional rates and long-term cycling via kinetically decoupled carbonization. Joule **9**(3), 101812 (2025). 10.1016/j.joule.2024.101812

[4] Y. Li, Q. Zhou, S. Weng, F. Ding, X. Qi et al., Interfacial engineering to achieve an energy density of over 200 Wh kg^−^^1^ in sodium batteries. Nat. Energy **7**(6), 511–519 (2022). 10.1038/s41560-022-01033-6

[5] D. Wang, Y. Chao, K. Guo, Z. Wang, M. Yang et al., Engineering metal electron spin polarization to regulate p-band center of Se for enhanced sodium-ion storage. Adv. Funct. Mater. **34**(40), 2405642 (2024). 10.1002/adfm.202405642

[6] W. Wang, Y. Shi, P. Li, R. Wang, F. Ye et al., Rational rock-salt phase engineering of a nickel-rich layered cathode interface for enhanced rate and cycling stability. Energy Environ. Sci. **17**(12), 4283–4294 (2024). 10.1039/D3EE04110G

[7] Y.-J. Guo, R.-X. Jin, M. Fan, W.-P. Wang, S. Xin et al., Sodium layered oxide cathodes: properties, practicality and prospects. Chem. Soc. Rev. **53**(15), 7828–7874 (2024). 10.1039/d4cs00415a 38962926 10.1039/d4cs00415a

[8] S.D. Shraer, N.D. Luchinin, I.A. Trussov, D.A. Aksyonov, A.V. Morozov et al., Development of vanadium-based polyanion positive electrode active materials for high-voltage sodium-based batteries. Nat. Commun. **13**, 4097 (2022). 10.1038/s41467-022-31768-5 35835761 10.1038/s41467-022-31768-5 PMC9283384

[9] T. Jin, H. Li, K. Zhu, P.-F. Wang, P. Liu et al., Polyanion-type cathode materials for sodium-ion batteries. Chem. Soc. Rev. **49**(8), 2342–2377 (2020). 10.1039/c9cs00846b 32222751 10.1039/c9cs00846b

[10] J. Peng, W. Zhang, Q. Liu, J. Wang, S. Chou et al., Prussian blue analogues for sodium-ion batteries: past, present, and future. Adv. Mater. **34**(15), 2108384 (2022). 10.1002/adma.202108384 10.1002/adma.202108384 34918850

[11] W. Wang, Y. Gang, Z. Hu, Z. Yan, W. Li et al., Reversible structural evolution of sodium-rich rhombohedral Prussian blue for sodium-ion batteries. Nat. Commun. **11**, 980 (2020). 10.1038/s41467-020-14444-4 32080172 10.1038/s41467-020-14444-4 PMC7033191

[12] Y. Zhang, S.-W. Zhang, Y. Chu, J. Zhang, H. Xue et al., Redefining closed pores in carbons by solvation structures for enhanced sodium storage. Nat. Commun. **16**, 3634 (2025). 10.1038/s41467-025-59022-8 40240373 10.1038/s41467-025-59022-8 PMC12003850

[13] S. Gan, Y. Huang, N. Hong, Y. Zhang, B. Xiong et al., Comprehensive understanding of closed pores in hard carbon anode for high-energy sodium-ion batteries. Nano-Micro Lett. **17**(1), 325 (2025). 10.1007/s40820-025-01833-x 10.1007/s40820-025-01833-x PMC12234992 40622590

[14] M. Liu, Z. Jiang, X. Wu, F. Liu, W. Li et al., Reinventing the high-rate energy storage of hard carbon: the order-degree governs the trade-off of desolvation-solid electrolyte interphase at interfaces. Angew. Chem. Int. Ed. **64**(17), e202425507 (2025). 10.1002/anie.202425507 10.1002/anie.202425507 39909841

[15] D. Chen, W. Zhang, K. Luo, Y. Song, Y. Zhong et al., Hard carbon for sodium storage: mechanism and optimization strategies toward commercialization. Energy Environ. Sci. **14**(4), 2244–2262 (2021). 10.1039/D0EE03916K

[16] X. Chen, N. Sawut, K. Chen, H. Li, J. Zhang et al., Filling carbon: a microstructure-engineered hard carbon for efficient alkali metal ion storage. Energy Environ. Sci. **16**(9), 4041–4053 (2023). 10.1039/d3ee01154b

[17] F. Wang, T. Zhang, T. Zhang, T. He, F. Ran, Recent progress in improving rate performance of cellulose-derived carbon materials for sodium-ion batteries. Nano-Micro Lett. **16**(1), 148 (2024). 10.1007/s40820-024-01351-2 10.1007/s40820-024-01351-2 PMC10928064 38466498

[18] G. Feng, X. Yang, X. Liu, Y. Wang, Y. Xie et al., Microbially glycolysis-regulated hard carbons for sodium-ion batteries. Nano Energy **136**, 110728 (2025). 10.1016/j.nanoen.2025.110728

[19] Y. Zeng, J. Yang, H. Yang, Y. Yang, J. Zhao, Bridging microstructure and sodium-ion storage mechanism in hard carbon for sodium ion batteries. ACS Energy Lett. **9**(3), 1184–1191 (2024). 10.1021/acsenergylett.3c02751

[20] K.-Y. Zhang, H.-H. Liu, J.-M. Cao, J.-L. Yang, M.-Y. Su et al., Microstructure reconstruction via confined carbonization achieves highly available sodium ion diffusion channels in hard carbon. Energy Storage Mater. **73**, 103839 (2024). 10.1016/j.ensm.2024.103839

[21] Z. Tang, R. Zhang, H. Wang, S. Zhou, Z. Pan et al., Revealing the closed pore formation of waste wood-derived hard carbon for advanced sodium-ion battery. Nat. Commun. **14**(1), 6024 (2023). 10.1038/s41467-023-39637-5 37758706 10.1038/s41467-023-39637-5 PMC10533848

[22] X. Chen, J. Tian, P. Li, Y. Fang, Y. Fang et al., An overall understanding of sodium storage behaviors in hard carbons by an adsorption-intercalation/filling hybrid mechanism. Adv. Energy Mater. **12**(24), 2200886 (2022). 10.1002/aenm.202200886

[23] N. Sun, J. Qiu, B. Xu, Understanding of sodium storage mechanism in hard carbons: ongoing development under debate. Adv. Energy Mater. **12**(27), 2200715 (2022). 10.1002/aenm.202200715

[24] S. Huang, Z. Li, B. Wang, J. Zhang, Z. Peng et al., N-doping and defective nanographitic domain coupled hard carbon nanoshells for high performance lithium/sodium storage. Adv. Funct. Mater. **28**(10), 1706294 (2018). 10.1002/adfm.201706294

[25] C. Wu, Y. Yang, Y. Li, X. He, Y. Zhang et al., Unraveling the structure–performance relationship in hard carbon for sodium-ion battery by coupling key structural parameters. Energy Environ. Sci. **18**(12), 6019–6031 (2025). 10.1039/d5ee00278h

[26] C. Qiu, A. Li, D. Qiu, Y. Wu, Z. Jiang et al., One-step construction of closed pores enabling high plateau capacity hard carbon anodes for sodium-ion batteries: closed-pore formation and energy storage mechanisms. ACS Nano **18**(18), 11941–11954 (2024). 10.1021/acsnano.4c02046 38652811 10.1021/acsnano.4c02046

[27] X.-X. He, W.-H. Lai, Y. Liang, J.-H. Zhao, Z. Yang et al., Achieving all-plateau and high-capacity sodium insertion in topological graphitized carbon. Adv. Mater. **35**(40), 2302613 (2023). 10.1002/adma.202302613 10.1002/adma.202302613 37390487

[28] J. Peng, H. Wang, X. Shi, H.J. Fan, Ultrahigh plateau-capacity sodium storage by plugging open pores. Adv. Mater. **37**(46), 2410326 (2025). 10.1002/adma.202410326 10.1002/adma.202410326 39604222

[29] Z. Zheng, S. Hu, W. Yin, J. Peng, R. Wang et al., CO_2_-etching creates abundant closed pores in hard carbon for high-plateau-capacity sodium storage. Adv. Energy Mater. **14**(3), 2303064 (2024). 10.1002/aenm.202303064

[30] S. You, Q. Zhang, J. Liu, Q. Deng, Z. Sun et al., Hard carbon with an opened pore structure for enhanced sodium storage performance. Energy Environ. Sci. **17**(21), 8189–8197 (2024). 10.1039/D4EE02519A

[31] L. Kitsu Iglesias, E.N. Antonio, T.D. Martinez, L. Zhang, Z. Zhuo et al., Revealing the sodium storage mechanisms in hard carbon pores. Adv. Energy Mater. **13**(44), 2302171 (2023). 10.1002/aenm.202302171

[32] J.C. Hyun, H.M. Jin, J.H. Kwak, S. Ha, D.H. Kang et al., Design guidelines for a high-performance hard carbon anode in sodium ion batteries. Energy Environ. Sci. **17**(8), 2856–2863 (2024). 10.1039/D4EE00315B

[33] J. Zhao, X.-X. He, W.-H. Lai, Z. Yang, X.-H. Liu et al., Catalytic defect-repairing using manganese ions for hard carbon anode with high-capacity and high-initial-coulombic-efficiency in sodium-ion batteries. Adv. Energy Mater. **13**(18), 2300444 (2023). 10.1002/aenm.202300444

[34] Y. Li, Y. Lu, Q. Meng, A.C.S. Jensen, Q. Zhang et al., Regulating pore structure of hierarchical porous waste cork-derived hard carbon anode for enhanced Na storage performance. Adv. Energy Mater. **9**(48), 1902852 (2019). 10.1002/aenm.201902852

[35] B. Jache, P. Adelhelm, Use of graphite as a highly reversible electrode with superior cycle life for sodium-ion batteries by making use of co-intercalation phenomena. Angew. Chem. Int. Ed. **53**(38), 10169–10173 (2014). 10.1002/anie.201403734 10.1002/anie.201403734 25056756

[36] Y. Li, A. Vasileiadis, Q. Zhou, Y. Lu, Q. Meng et al., Origin of fast charging in hard carbon anodes. Nat. Energy **9**(2), 134–142 (2024). 10.1038/s41560-023-01414-5

[37] G. Kresse, J. Furthmüller, Efficiency of ab-initio total energy calculations for metals and semiconductors using a plane-wave basis set. Comput. Mater. Sci. **6**(1), 15–50 (1996). 10.1016/0927-0256(96)00008-0 10.1103/physrevb.54.11169 9984901

[38] G. Kresse, J. Furthmüller, Efficient iterative schemes for ab initio total-energy calculations using a plane-wave basis set. Phys. Rev. B **54**(16), 11169–11186 (1996). 10.1103/physrevb.54.11169 10.1103/physrevb.54.11169 9984901

[39] J.P. Perdew, K. Burke, M. Ernzerhof, Generalized gradient approximation made simple. Phys. Rev. Lett. **77**(18), 3865–3868 (1996). 10.1103/physrevlett.77.3865 10062328 10.1103/PhysRevLett.77.3865

[40] P.E. Blöchl, Projector augmented-wave method. Phys. Rev. B **50**(24), 17953–17979 (1994). 10.1103/physrevb.50.17953 10.1103/physrevb.50.17953 9976227

[41] S. Grimme, Semiempirical GGA-type density functional constructed with a long-range dispersion correction. J. Comput. Chem. **27**(15), 1787–1799 (2006). 10.1002/jcc.20495 16955487 10.1002/jcc.20495

[42] H.J. Monkhorst, J.D. Pack, Special points for Brillouin-zone integrations. Phys. Rev. B **13**(12), 5188–5192 (1976). 10.1103/physrevb.13.5188

[43] A. Van der Ven, M.K. Aydinol, G. Ceder, G. Kresse, J. Hafner, First-principles investigation of phase stability in Li_*x*_CoO_2_. Phys. Rev. B **58**(6), 2975–2987 (1998). 10.1103/physrevb.58.2975

[44] M. Wagemaker, A. Van Der Ven, D. Morgan, G. Ceder, F.M. Mulder et al., Thermodynamics of spinel Lix TiO_2_ from first principles. Chem. Phys. **317**(2–3), 130–136 (2005). 10.1016/j.chemphys.2005.05.011

[45] M.K. Aydinol, A.F. Kohan, G. Ceder, K. Cho, J. Joannopoulos, Ab initio study of lithium intercalation in metal oxides and metal dichalcogenides. Phys. Rev. B **56**(3), 1354–1365 (1997). 10.1103/physrevb.56.1354

[46] W.G. Hoover, Canonical dynamics: equilibrium phase-space distributions. Phys. Rev. A **31**(3), 1695–1697 (1985). 10.1103/physreva.31.1695 10.1103/physreva.31.1695 9895674

[47] S. Nosé, A unified formulation of the constant temperature molecular dynamics methods. J. Chem. Phys. **81**(1), 511–519 (1984). 10.1063/1.447334

[48] W. Humphrey, A. Dalke, K. Schulten, VMD: visual molecular dynamics. J. Mol. Graph. **14**(1), 33–38 (1996). 10.1016/0263-7855(96)00018-5 8744570 10.1016/0263-7855(96)00018-5

[49] X. Sun, X. Zhang, Q. Ma, X. Guan, W. Wang et al., Revisiting the electroplating process for lithium-metal anodes for lithium-metal batteries. Angew. Chem. Int. Ed. **59**(17), 6665–6674 (2020). 10.1002/anie.201912217 10.1002/anie.201912217 31587466

[50] F. Huang, P. Xu, G. Fang, S. Liang, In-depth understanding of interfacial Na^+^ behaviors in sodium metal anode: migration, desolvation, and deposition. Adv. Mater. **36**(41), 2405310 (2024). 10.1002/adma.202405310 10.1002/adma.202405310 39152941

[51] M.P. Mercer, S. Affleck, E.M. Gavilán-Arriazu, A.A. Zülke, P.A. Maughan et al., Sodiation of hard carbon: how separating enthalpy and entropy contributions can find transitions hidden in the voltage profile. ChemPhysChem **23**(5), e202100748 (2022). 10.1002/cphc.202100748 34859948 10.1002/cphc.202100748

[52] M.P. Mercer, M. Nagarathinam, E.M. Gavilán-Arriazu, A. Binjrajka, S. Panda et al., Sodiation energetics in pore size controlled hard carbons determined via entropy profiling. J. Mater. Chem. A **11**(12), 6543–6555 (2023). 10.1039/D2TA09406A

[53] D. Kim, K. Zhang, M. Cho, Y.-M. Kang, Critical design factors for kinetically favorable P-based compounds toward alloying with Na ions for high-power sodium-ion batteries. Energy Environ. Sci. **12**(4), 1326–1333 (2019). 10.1039/C9EE00283A

[54] S.C. Dey, B. Worfolk, L. Lower, W.J. Sagues, M.R. Nimlos et al., Phenolic resin derived hard carbon anode for sodium-ion batteries: a review. ACS Energy Lett. **9**(6), 2590–2614 (2024). 10.1021/acsenergylett.4c00688

[55] X. Yin, Z. Lu, J. Wang, X. Feng, S. Roy et al., Enabling fast Na+ transfer kinetics in the whole-voltage-region of hard-carbon anodes for ultrahigh-rate sodium storage. Adv. Mater. **34**(13), 2109282 (2022). 10.1002/adma.202109282 10.1002/adma.202109282 35075693

[56] Y. Chu, J. Zhang, Y. Zhang, Q. Li, Y. Jia et al., Reconfiguring hard carbons with emerging sodium-ion batteries: a perspective. Adv. Mater. **35**(31), 2212186 (2023). 10.1002/adma.202212186 10.1002/adma.202212186 36806260

[57] X. Li, J. Zhang, J. Zhang, L. Guo, H. Zhang et al., C_60_ to modulate the closed pore structures of hard carbon for high-performance sodium-ion batteries. ACS Nano **19**(15), 14829–14838 (2025). 10.1021/acsnano.4c18421 40219987 10.1021/acsnano.4c18421

[58] S. Xiao, Y.-J. Guo, H.-X. Chen, H. Liu, Z.-Q. Lei et al., Insight into the role of closed-pore size on rate capability of hard carbon for fast-charging sodium-ion batteries. Adv. Mater. **37**(28), 2501434 (2025). 10.1002/adma.202501434 10.1002/adma.202501434 40244711

[59] P. Veerakumar, S. Manavalan, S.-M. Chen, A. Pandikumar, K.-C. Lin, Ultrafine Bi–Sn nanoparticles decorated on carbon aerogels for electrochemical simultaneous determination of dopamine (neurotransmitter) and clozapine (antipsychotic drug). Nanoscale **12**(43), 22217–22233 (2020). 10.1039/D0NR06028C 33141140 10.1039/d0nr06028c

[60] D. Wu, F. Sun, Z. Qu, H. Wang, Z. Lou et al., Multi-scale structure optimization of boron-doped hard carbon nanospheres boosting the plateau capacity for high performance sodium ion batteries. J. Mater. Chem. A **10**(33), 17225–17236 (2022). 10.1039/D2TA04194D

[61] Z. Jian, Z. Xing, C. Bommier, Z. Li, X. Ji, Hard carbon microspheres: potassium-ion anode versus sodium-ion anode. Adv. Energy Mater. **6**(3), 1501874 (2016). 10.1002/aenm.201501874

[62] K. Wang, F. Sun, H. Wang, D. Wu, Y. Chao et al., Altering thermal transformation pathway to create closed pores in coal-derived hard carbon and boosting of Na^+^ plateau storage for high-performance sodium-ion battery and sodium-ion capacitor. Adv. Funct. Mater. **32**(34), 2203725 (2022). 10.1002/adfm.202203725

[63] H.G.T. Nguyen, J.C. Horn, M. Bleakney, D.W. Siderius, L. Espinal, Understanding material characteristics through signature traits from helium pycnometry. Langmuir **35**(6), 2115–2122 (2019). 10.1021/acs.langmuir.8b03731 30698443 10.1021/acs.langmuir.8b03731 PMC6594170

[64] J. Yang, X. Wang, W. Dai, X. Lian, X. Cui et al., From micropores to ultra-micropores inside hard carbon: toward enhanced capacity in room-/ low-temperature Sodium-ion storage. Nano-Micro Lett. **13**(1), 98 (2021). 10.1007/s40820-020-00587-y 10.1007/s40820-020-00587-y PMC8010088 34138264

[65] L. Liu, Y. Gu, J. Li, S. Bashir, R. Kasi et al., A stage-wise plateau-sodiation mechanism enabled by ultramicropores in the hard carbon anode for sodium storage. Adv. Energy Mater. **16**(4), e04853 (2026). 10.1002/aenm.202504853

[66] J. Lin, Q. Zhou, Z. Liao, Y. Chen, Y. Liu et al., Steric hindrance engineering to modulate the closed pores formation of polymer-derived hard carbon for high-performance Sodium-ion batteries. Angew. Chem. Int. Ed. **63**(39), e202409906 (2024). 10.1002/anie.202409906 10.1002/anie.202409906 38970247

[67] L. Bai, J. Chen, Y. Zhang, Z. Yi, Y. Xie et al., Oxygen-bridging and air-etching: advanced pore-regulation engineering in hard carbons for optimized low-voltage plateau capacity. Nano Energy **135**, 110692 (2025). 10.1016/j.nanoen.2025.110692

[68] Y. Qiu, Y. Su, X. Jing, H. Xiong, D. Weng et al., Rapid closed pore regulation of biomass-derived hard carbons based on flash joule heating for enhanced Sodium ion storage. Adv. Funct. Mater. **35**(29), 2423559 (2025). 10.1002/adfm.202423559

[69] J. Vermesse, D. Vidal, P. Malbrunot, Gas adsorption on zeolites at high pressure. Langmuir **12**(17), 4190–4196 (1996). 10.1021/la950283m

[70] Y. Liu, Z. Liu, J. Jia, G. Ye, Y. Xie et al., Engineering the dynamic hydrogen bonds in π-stacked supramolecular assemblies for hierarchical nanocrystal formation. Chem. Mater. **34**(7), 3525–3535 (2022). 10.1021/acs.chemmater.2c00595

[71] H. Kim, J. Hong, G. Yoon, H. Kim, K.-Y. Park et al., Sodium intercalation chemistry in graphite. Energy Environ. Sci. **8**(10), 2963–2969 (2015). 10.1039/c5ee02051d

[72] X. Liu, T. Wang, T. Zhang, Z. Sun, T. Ji et al., Solvated sodium storage via a coadsorptive mechanism in microcrystalline graphite fiber. Adv. Energy Mater. **12**(45), 2202388 (2022). 10.1002/aenm.202202388

[73] Z. Lu, H. Yang, Y. Guo, H. Lin, P. Shan et al., Consummating ion desolvation in hard carbon anodes for reversible sodium storage. Nat. Commun. **15**(1), 3497 (2024). 10.1038/s41467-024-47522-y 38664385 10.1038/s41467-024-47522-y PMC11045730

[74] Z. Liu, Y. Tian, S. Li, L. Wang, B. Han et al., Revealing high-rate and high volumetric pseudo-intercalation charge storage from boron-vacancy doped MXenes. Adv. Funct. Mater. **33**(40), 2301994 (2023). 10.1002/adfm.202301994

[75] Y. Chao, S. Jia, J. Li, G. Chen, L. Liu et al., A dual heterostructure enables the stabilization of 1T-rich MoSe_2_ for enhanced storage of sodium ions. Chem. Sci. **15**(28), 11134–11144 (2024). 10.1039/D4SC02400A 39027283 10.1039/d4sc02400a PMC11253150

[76] G. Dai, H. Xu, Y. Jiang, W. Gong, H. Sun et al., Harnessing 5d electron spin for asymmetric Ir–Zn atomic pairs toward efficient and durable sulfur conversion catalysis. Appl. Catal. B Environ. Energy **378**, 125608 (2025). 10.1016/j.apcatb.2025.125608

[77] Y. Wang, X. Xu, Y. Wu, F. Li, W. Fan et al., Facile galvanic replacement construction of Bi@C nanosheets array as binder-free anodes for superior sodium-ion batteries. Adv. Energy Mater. **14**(30), 2401833 (2024). 10.1002/aenm.202401833

[78] J. Zhu, J. Fan, T. Cheng, M. Cao, Z. Sun et al., Bilayer nanosheets of unusual stoichiometric bismuth oxychloride for potassium ion storage and CO_2_ reduction. Nano Energy **75**, 104939 (2020). 10.1016/j.nanoen.2020.104939

[79] C. Chen, Y. Tian, R. Ren, S. Duan, D. Wang et al., Regulating pores and carbonyl groups of biomass-derived hard carbon for enhanced sodium storage. Adv. Sci. **12**(40), e10328 (2025). 10.1002/advs.202510328 10.1002/advs.202510328 PMC12561183 40736458

[80] G. Dai, W. Gong, H. Sun, H. Liu, Y. Jiang et al., Activating inert metallic zinc for bifunctional sulfur reaction catalysis through anion-controlled tensile lattice strain. Adv. Energy Mater. **15**(48), e02123 (2025). 10.1002/aenm.202502123

