# Supplementary material for: Nano-Space Confinement Drives Rational Closed Pore Design in Hard Carbons for High-Capacity and High-Rate Sodium Storage
Source: Nanomicro Lett. 2026 May 21;18:382. doi: 10.1007/s40820-026-02223-7 (PMC13194839; doi:10.1007/s40820-026-02223-7)
Supplement: Supplementary file 7 — Supplementary file7 (DOCX 13371 KB) [file 40820_2026_2223_MOESM7_ESM.docx]

Supporting Information for

**Nano-Space Confinement Drives Rational Closed Pore Design in Hard Carbons for High-Capacity and High-Rate Sodium Storage**

Run Ren^1,2,†^, Ling Zhang^2,†^, Jianhua Zhu^1^,*, Yunfeng Chao^1^, Junlin Guo^3^, Yijun Cao^2^, Xiaobo Ji^4^, Xinwei Cui^1,3,5^*

^1^ Henan Institutes of Advanced Technology, Zhengzhou University, Zhengzhou 450003, P. R. China

^2^ Zhongyuan Critical Metals Laboratory, Zhengzhou University, Zhengzhou 450001, P. R. China

^3^ School of Materials Science and Engineering, Zhengzhou University, Zhengzhou 450001, P. R. China

^4^ College of Chemistry and Chemical Engineering, Central South University, Changsha 410083, P. R. China

^5^ State Key Laboratory of Coking Coal Resources Green Exploitation, Zhengzhou University, Zhengzhou 450001, P. R. China

^†^ Run Ren and Ling Zhang contribute equally to this work.

*Corresponding authors. E-mail: [xinweic@zzu.edu.cn](mailto:xinweic@zzu.edu.cn) (Xinwei Cui); [jianhuazhu@zzu.edu.cn](mailto:jianhuazhu@zzu.edu.cn) (Jianhua Zhu)

**Supporting Figures**


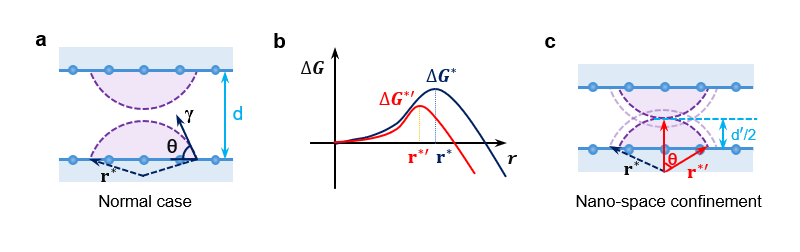


**Fig. S1** Theoretical derivative of the activation free energy () of Na clusters within the nanoconfined space related as a function of the interlayer spacing *d*.

According to heterogenous nucleation in (a)

$$\Delta G^{*}=(\frac{16\pi\gamma^{3}}{3\Delta H_{f}^{2}})\cdot(\frac{T_{m}}{\Delta T})^{2}\cdot(\frac{2-3\cos\theta+\cos^{3}\theta}{4})$$

$$r^{*}=-\frac{2\gamma}{\Delta G_{v}}$$

which are labeled in the blue curve of $\Delta G\sim r$ in (b). In addition, $\Delta G_{v}$ is volume free energy,

The units are J, J m^–2^, J m^–3^, K, K, rad, m, J m^–3^ for $\Delta G^{*}$, $\gamma$, $\Delta H_{f}$, $T_{m}$, $\Delta T$, $\theta$, $r^{*}$, $\Delta G_{v}$, respectively. As the size ($d$) of the closed pore decreases, $r^{*}$ decreases, then the new critical radius of the nucleus () can be related to $d'$. According to (c):

Thus,

Assuming that follows the condition given by the red line of $\Delta G\sim r$in (b), then,

thus,

then,

$$\frac{T_{m}}{\Delta T'}=-\frac{d'}{2(1-\cos\theta)}\cdot\frac{\Delta H_{f}}{2\gamma}$$

In addition,

Combining the above two equations, we obtain

Simplifying the equation above, we finally obtain

The units are J, m, m, m, K, J m^–3^ for , $d$, $d'$, , $\Delta T'$, $\Delta G_{v}^{'}$, respectively.

This equation suggests that as the available space becomes more confined, the critical nucleus size decreases, thereby favoring nucleation at lower energy compensation following the square law.


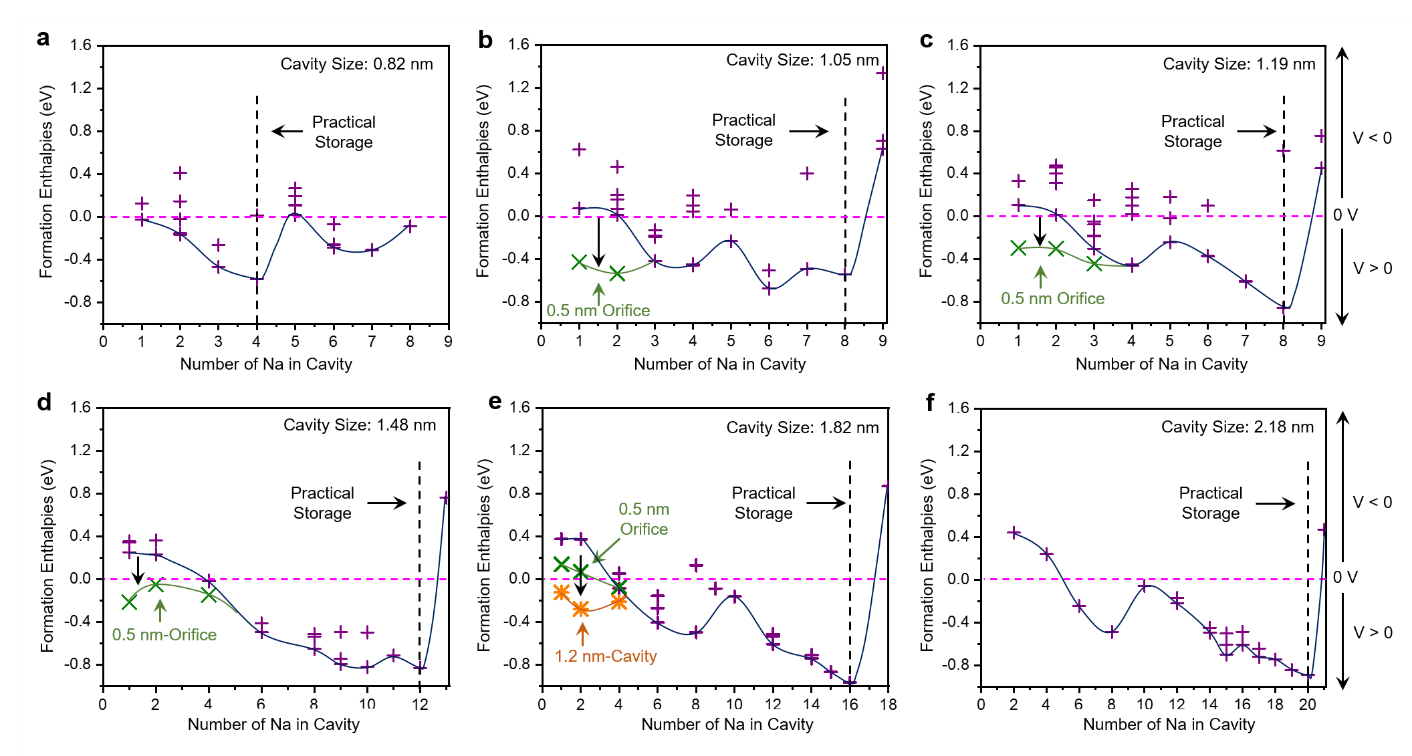


**Fig. S2** Formation enthalpy changes with the number of Na stored in six different cavity size. On the basis of the lowest enthalpy path, we can obtain formation enthalpy ($H$) profiles along with the Na sodiation process, where $H$<0 corresponds to $V$>0. Green and orange lines illustrate the case of pre-nucleation in order to reduce energy barrier.

The rationale behind the setup of the double-layer graphene models used for DFT calculations in Figs. 1 and S2: Firstly, we built sodiation models with increased layers of pure metallic Na within cavities. After optimization without fixation of graphitic walls, theoretical sodiation models can be determined, from which the interlayer spacings of 0.82 nm, 1.05 nm, 1.19 nm, 1.48 nm, 1.82 nm, and 2.18 nm were obtained. Secondly, with these cavity sizes fixed, the electrochemical processes were then simulated by filling the corresponding the graphitic cavities with Na atoms one by one in energy competing positions and performing geometrical and energy optimizations, from which the practical sodiation models can be determined. Although the closed pores may expand during sodiation, the fixed interlayer spacing in the second step calculation represents the maximum size a cavity can reach as it is inflated by a Na cluster. Therefore, this approach enables the establishment of a relationship between the maximum inflated cavity size and Na nucleation/growth within the cavity, thereby reasonably simplifying our discussion about Na-storage behavior in closed pores.


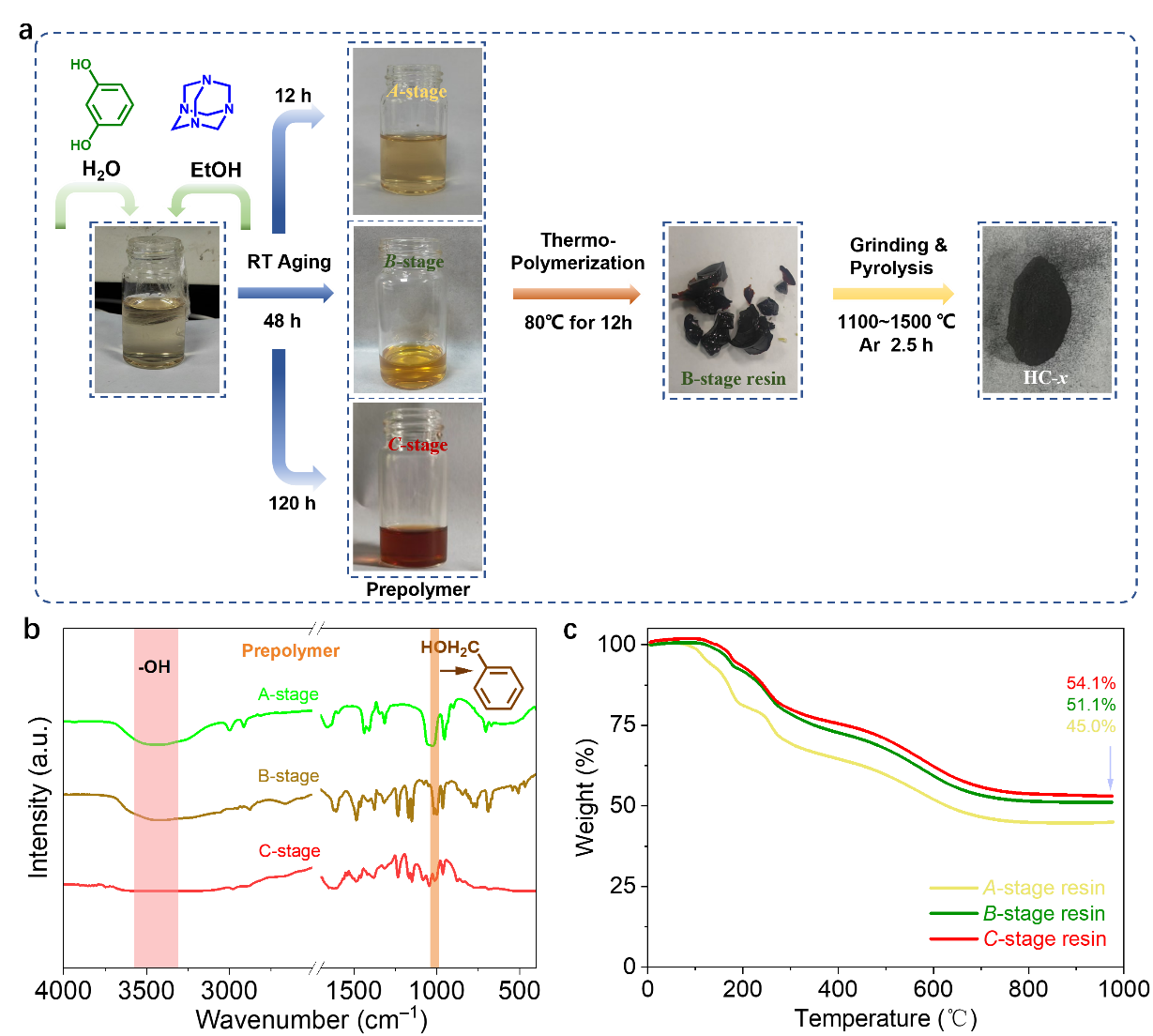


**Fig. S3** Characterization of prepolymer structures before thermo-polymerization and resins before pyrolysis. **a** Fabrication process of our resin-based HCs. No gelation forms after room-temperature aging, implying the supressed crosslinking during this step. **b** FTIR spectra of the prepolymer structures before thermo-polymerization, revealing the decreased amount of −CH_2_OH end groups and the growth of polymer chains with prolonged aging time. **c** Thermogravimetric analysis (TGA) data of three resins after thermos-polymerization, showing distinct mass loss patterns

**
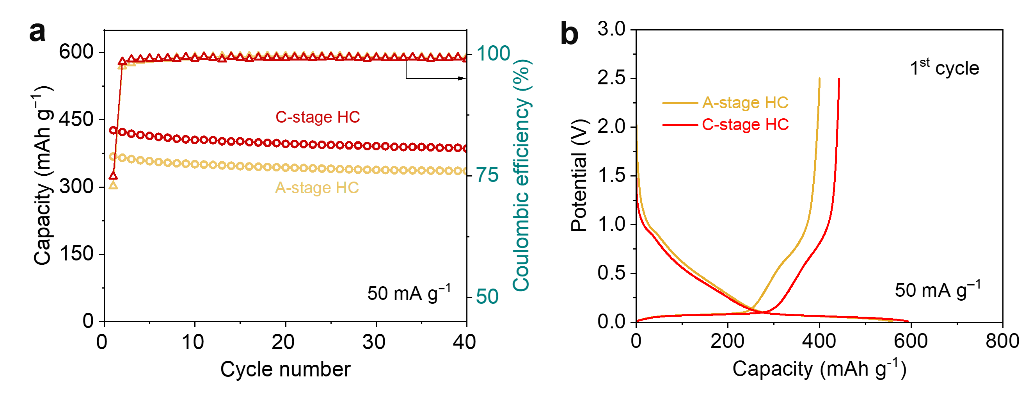
**

**Fig. S4** Electrochemical performance of A- and C-stage resin-derived HCs at 1300℃. **a** Cycling performance. **b** Charge-discharge curves at the first cycle

**
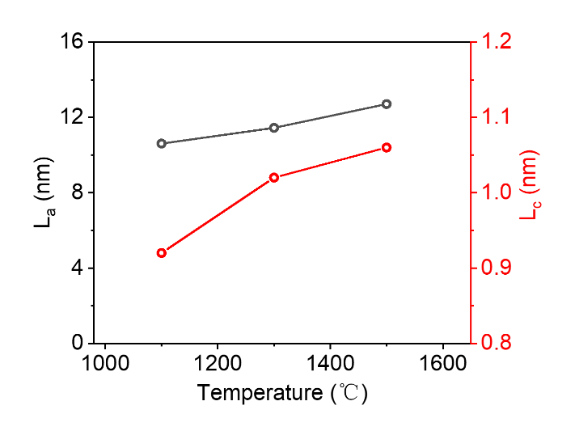
**

**Fig. S5** The average graphitic domain length (*L*_a_) and the graphitic crystallite thickness (*L*_c_) content versus pyrolysis temperatures, derived from XRD patterns in Fig. 2b. *L*_a_ and *L*_c_ can be calculated via the Scherrer equations of $L_{a}=1.84\lambda/B_{100}\cos\theta_{100}$ and $L_{c}=0.89\lambda/B_{002}\cos\theta_{002}$,[S1] where λ is the wavelength of the X-rays (0.154 nm), *B*_100_ and *B*_002_ are the full width at half-maxima of the (100) and (002) peaks, and 2*θ*_100_ and 2*θ*_002_ are the corresponding peak positions.

**
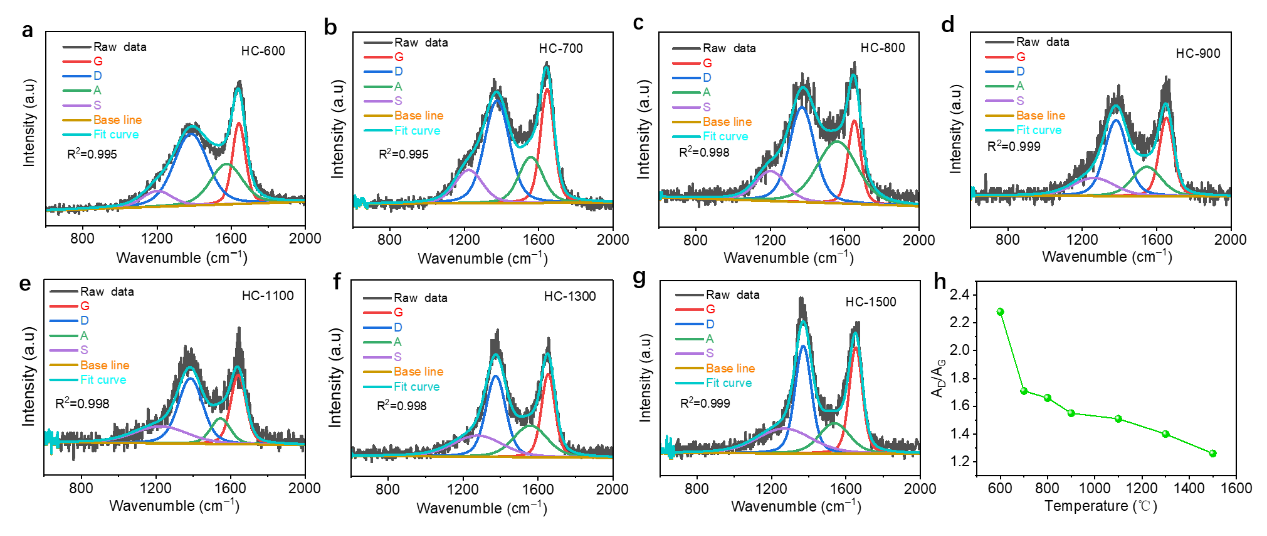
**

**Fig. S6** Raman spectra of HCs. **a-g** Fitted Raman patterns of B-stage resin-derived HCs pyrolyzed at different temperatures ranging from 600°C to 1500°C. **h** A_D_/A_G_ as a function of pyrolysis temperature, indicating a decrease in structural defects with increasing pyrolysis temperature

To get accurate information, the Raman spectra were divided into four bands, ie., S-, D-, A-, and G-bands, according to previous studies.[S2] The S band (purple) located at 1100–1200 cm^−1^ is attributed to rings containing 7+ carbons, and the A band (green) between the D (blue) and G (red) can be assigned to vacancy point defects. Therefore, the ratio between the band areas, A_D_/A_G_ ratio, is used to estimate the defect concentration, as demonstrated in (**h**).

**
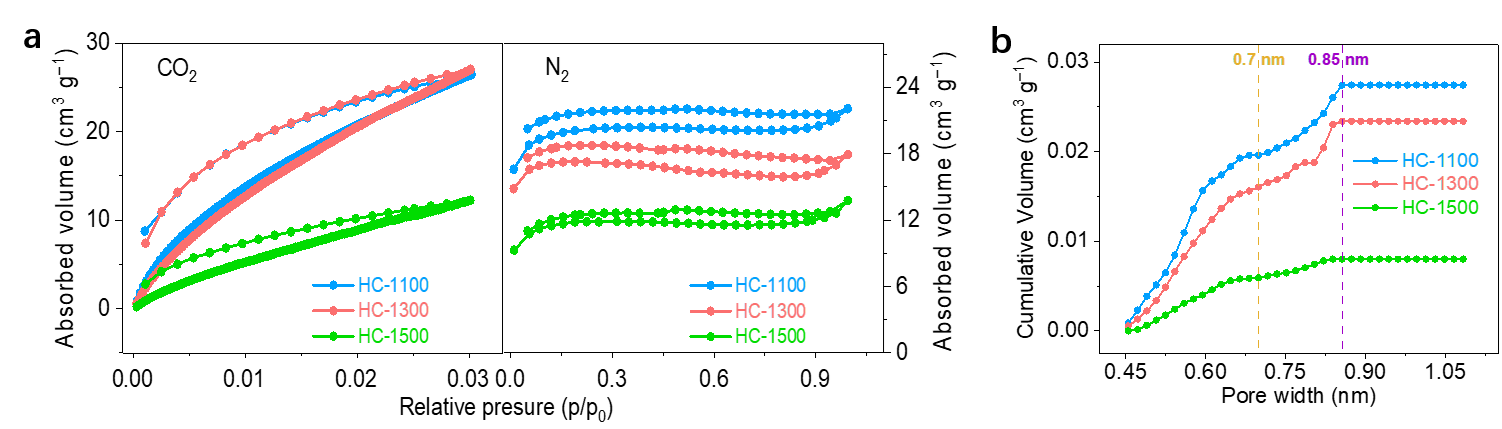
**

**Fig. S7** CO_2_ and N_2_ gas physisorption analyses showing the open pore structures of three different HCs. **a** Adsorption-desorption isotherms. **b** Cumulative volume versus pore width

**
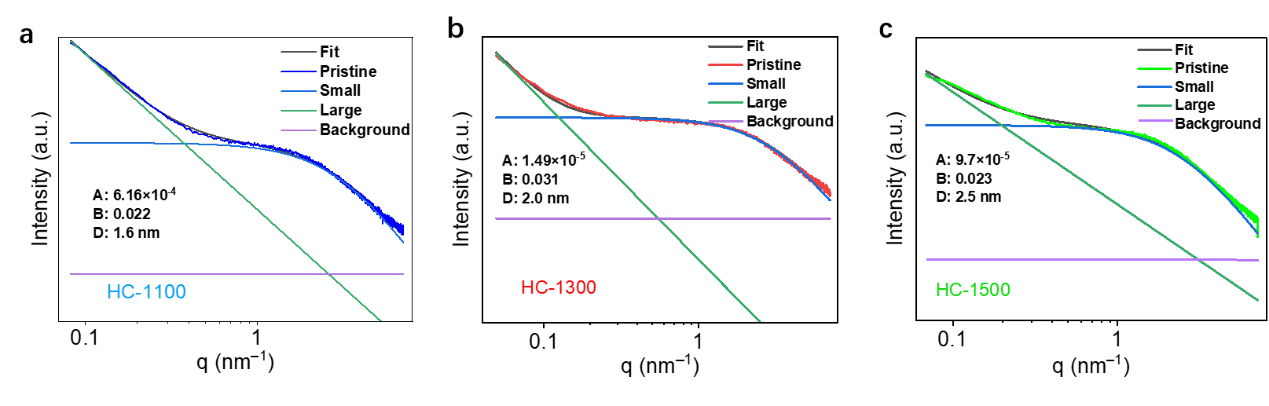
**

**Fig. S8** Fitted SAXS patterns of (**a**) HC-1100, (**b**) HC-1300, and (**c**) HC-1500. The patterns are fitted based on the following model [S3]:

$$I(Q)=\frac{A}{Q^{\alpha}}+\frac{Ba_{1}^{4}}{(1+a_{1}^{2}Q^{2})^{2}}+D$$

*A* is a coefficient of porod theory while *B* is positively correlated with the number of pores and specific surface area. The fractal dimension $\alpha$ characterizes the material’s hierarchical structure. Parameter *D* represents a constant background term. The diameter of closed nanopores can be calculated using the following empirical relationship:

$$d=2\times a_{1}\times10^{1/2}$$

**
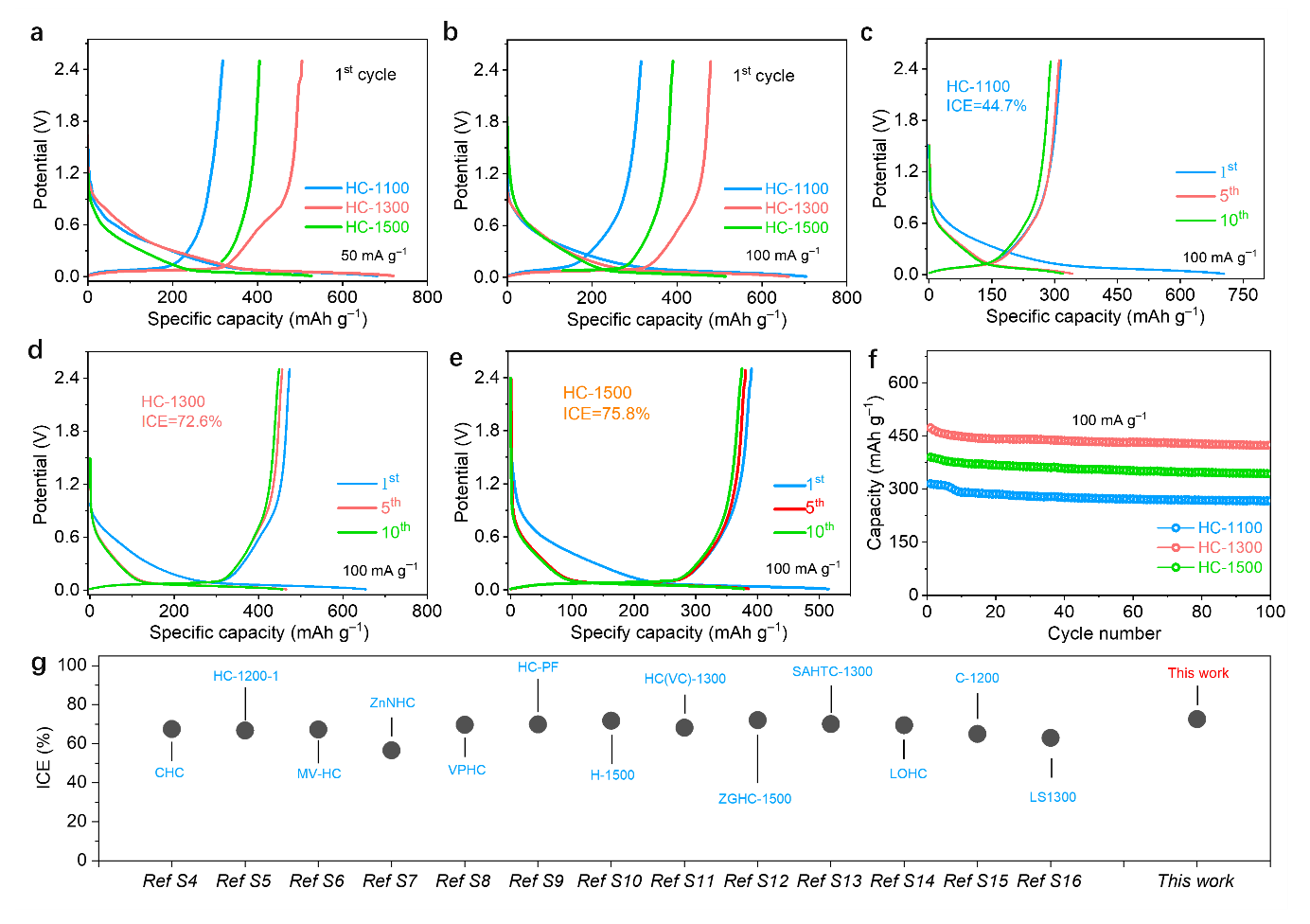
**

**Fig. S9** Additional electrochemical performance of HC-1100, HC-1300, and HC-1500 electrodes. **a,b** Charge-discharge profiles of HCs for the first cycle at (**a**) 50 mA g^−1^ and (**b**) 100 mA g^−1^. **c-e** Charge-discharge profiles of HCs for the first 10 cycles at 100 mA g^−1^. **f** Cycling performance of HCs at 100 mA g^−1^. **g**, Comparison of ICE vales with reported HC anodes for SIBs [S4-S16]


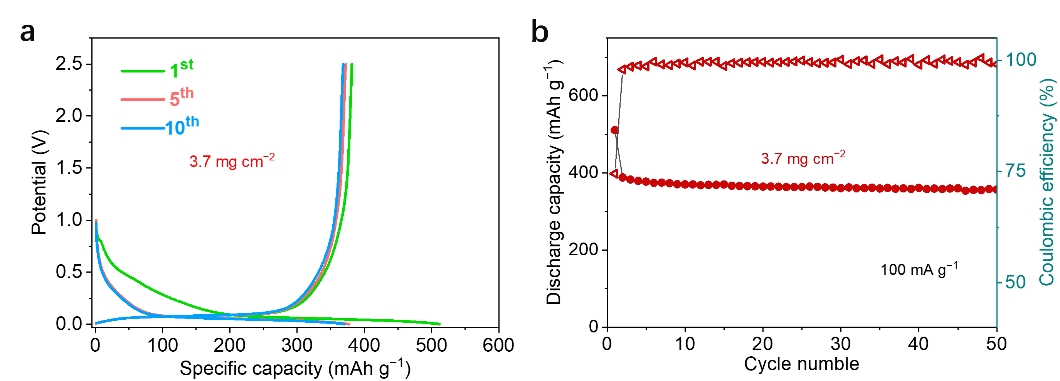


**Fig. S10** Electrochemical performance of HC-1300 electrodes with a high areal loading of 3.7 mg cm^−2^. **a**, GCD profiles. **b**, Cycling performance


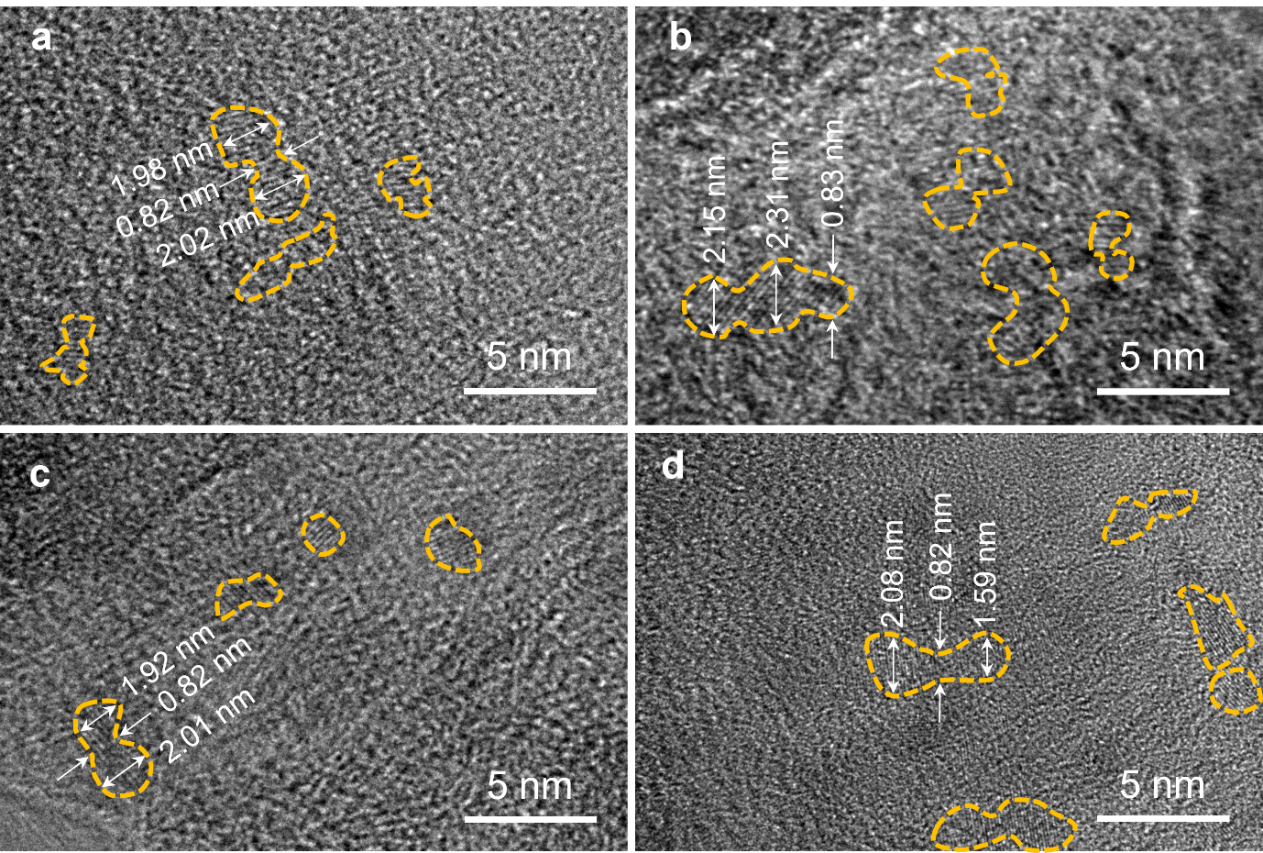


**Fig. S11** HRTEM images of HC-1300 after being discharged to the fully sodiated state at 0.01 V

**
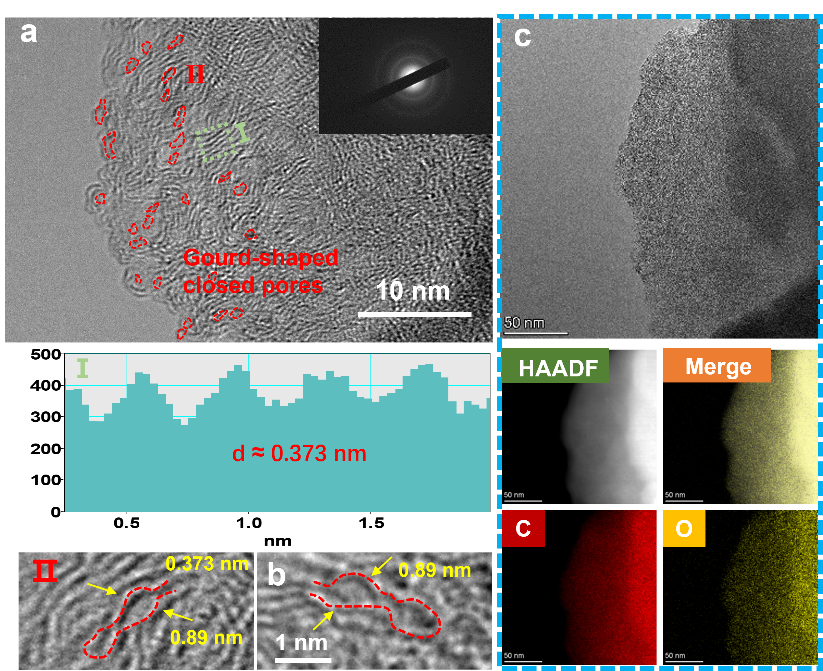
**

**Fig. S12** TEM analyses of the synthesized HC-1300 before sodiation. **a**,**b** TEM and HRTEM images. **c** TEM image and corresponding mapping images of HC-1300

**
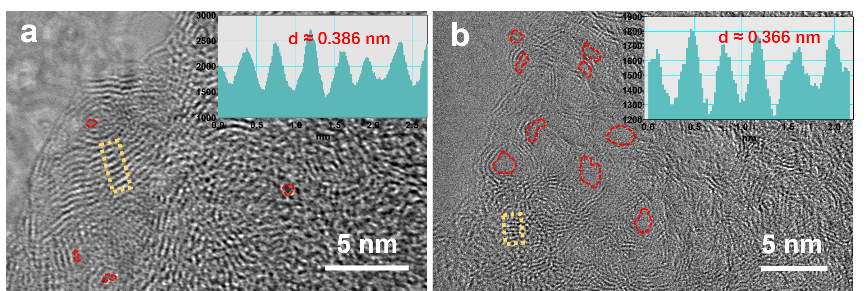
**

**Fig. S13** HRTEM analyses of the synthesized HC-1100 and HC-1500 before sodiation. **a** HC-1100 and **b** HC-1500

**
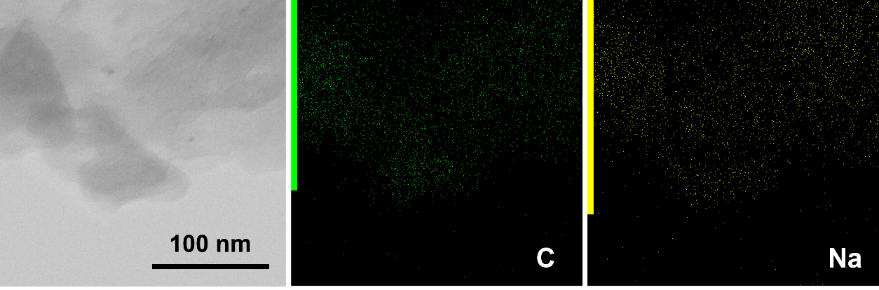
**

**Fig. S14** Elemental mapping analysis of HC-1300 in the sodiated state. HAADF and the corresponding EDS elemental mappings of C and Na in HC-1300 after discharge to 0.01 V


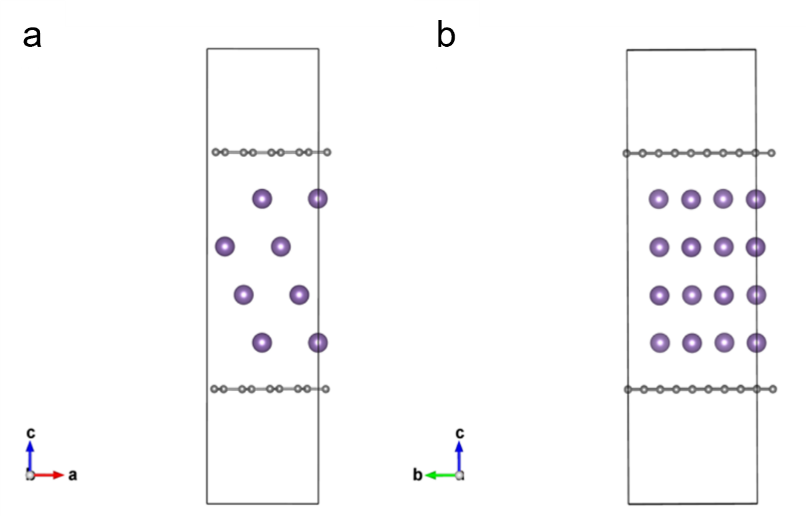


**Fig. S15** The optimized sodiation structure practically stored in a cavity size of 1.82 nm and viewed in two different directions


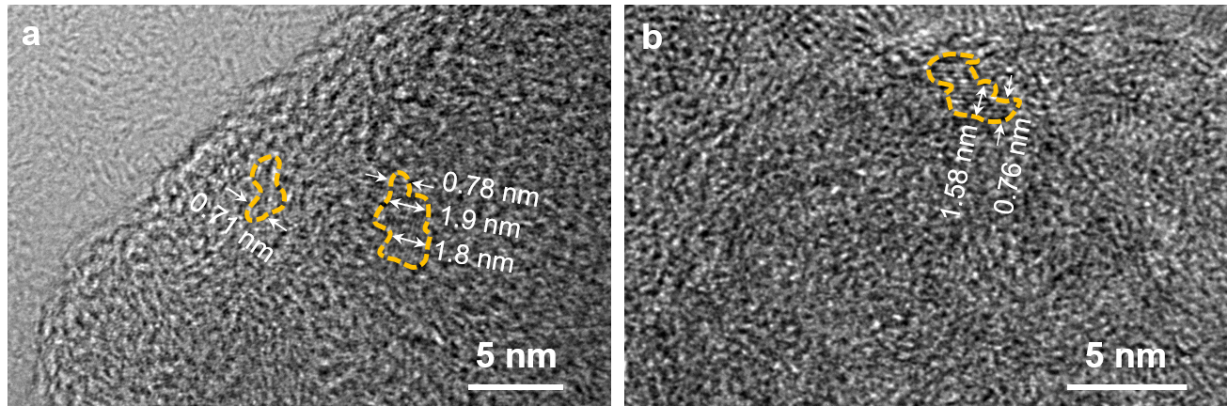


**Fig. S16** HRTEM images of HC-1100 after being discharged to the fully sodiated state at 0.01 V. Very few and small stage-wise Na clusters can be seen


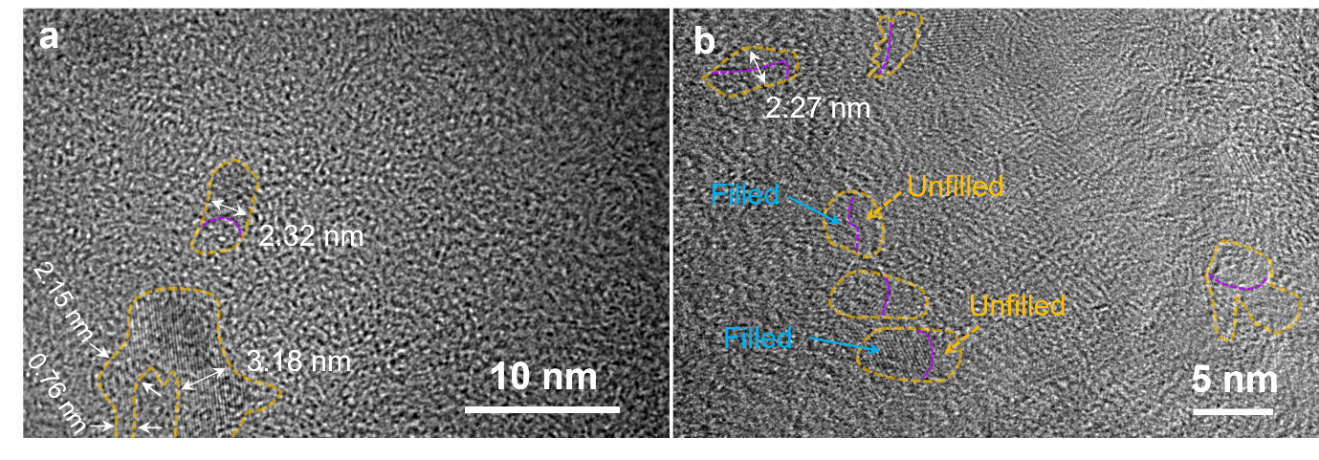


**Fig. S17** HRTEM images of HC-1500 after discharged to the fully sodiated state at 0.01 V. **a,b** A large Na cluster with a four-stage closed pore structure is shown in (**a**), while most of the pores are incompletely filled, as indicated in (**a**) and (**b**)


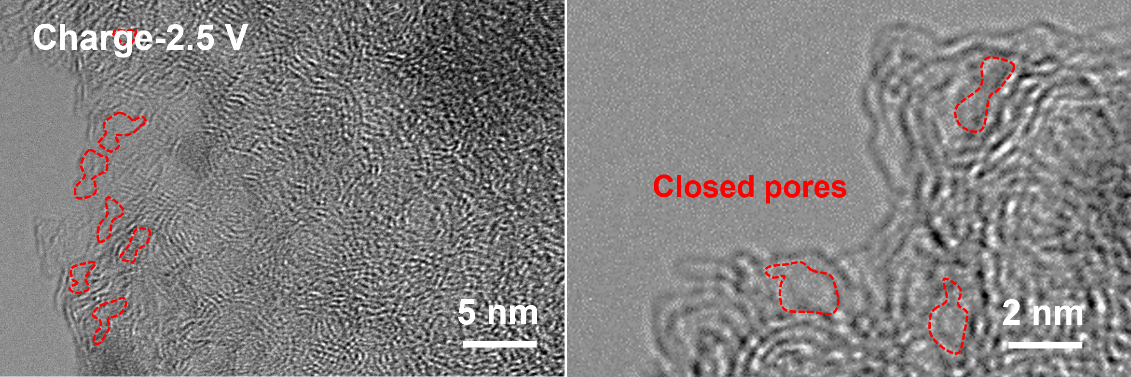


**Fig. S18** HRTEM images of HC-1300 after 50 cycles at 0.2 A g^–1^

**
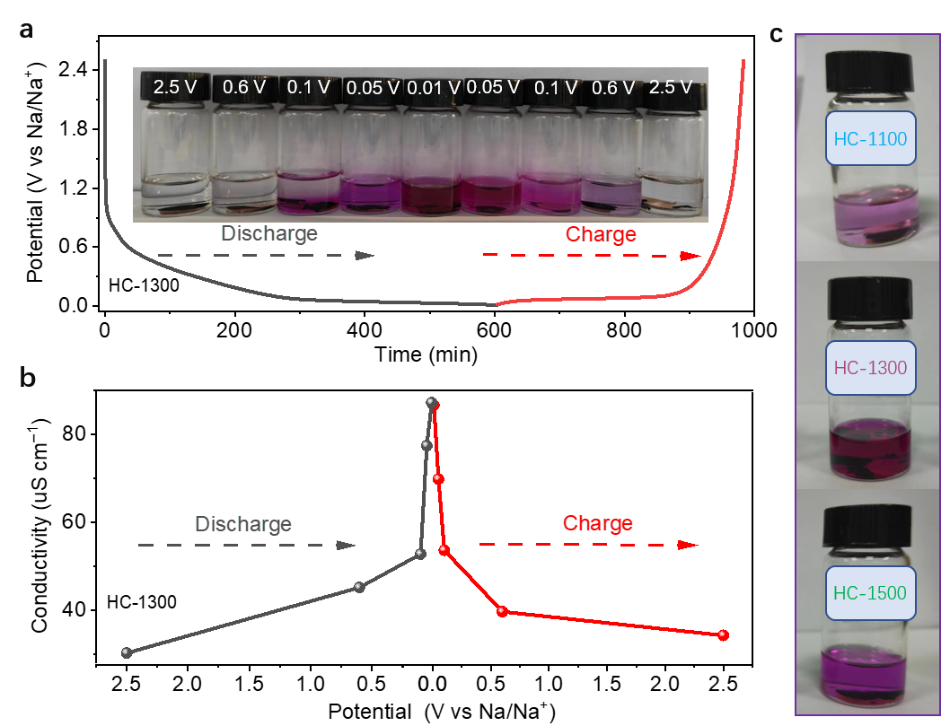
**

**Fig. S19** Qualitative determination of quasi-metallic Na confined within closed pores of HC anodes. **a** Typical charge–discharge profile of the HC-1300 electrode and corresponding color changes of ethanol containing 1% phenolphthalein after reaction with HC-1300 at different potentials. **b** Ionic conductivity of the ethanol solutions after reaction with HC-1300 at various potentials. **c** Color comparison of 1% phenolphthalein ethanol solutions after reaction with three HCs at the fully discharged state of 0.01 V

Through the utilization of specific reactions between sodiated HC electrodes and ethanol, the quasi-metallic nature of Na and its relative content in the three HCs can be corroborated. First, Movies S1-S3 distinctly capture the reaction phenomena between three fully-discharged HC electrodes and ethanol: rapid bubble generation accompanied by intense red coloration of phenolphthalein solution, with the HC-1300 electrode exhibiting the most rapid reaction rate and deepest phenolphthalein coloration. Second, after reacting with HC-1300 electrodes, the ethanol solution progressively darkens during discharge and lightens during charging (Fig. S19a), and the ionic conductivity of the corresponding solutions increases progressively during discharge and returns to the original level after charging back to 2.5 V (Fig. S19b). The coloration intensity trend of HC-1300 > HC-1500 > HC-1100 in Figure S19c aligns well with the results shown in Movies S1-3, confirming that the fully-discharged HC-1300 electrode contains the highest amount of quasi-metallic Na clusters stored in closed pores at 0.01 V.


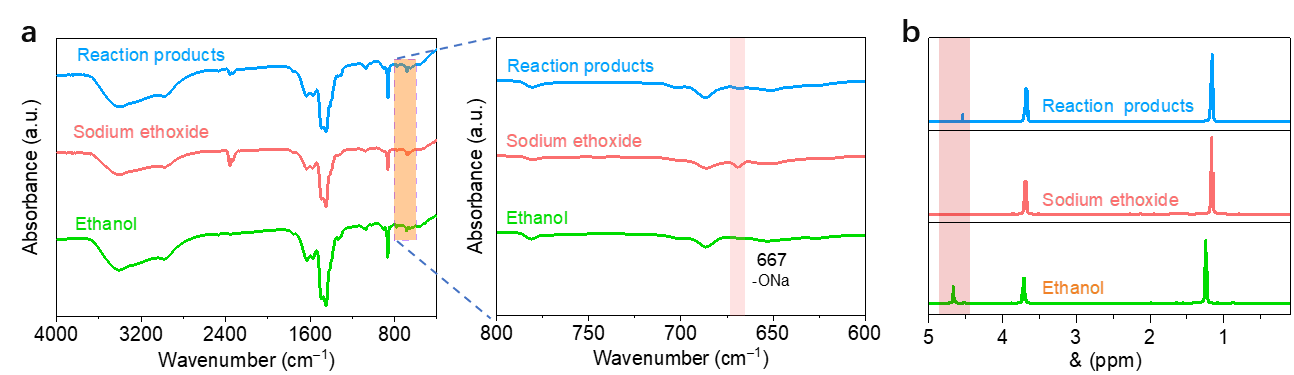


**Fig. S20** Characterization of the products of the reaction between the fully sodiated HC-1300 electrode and ethanol, in comparison with pure ethanol solution and sodium ethoxide. **a,b** FTIR spectra. **c** ^1^H NMR spectra, using CDCl_3_ as the standard solution. It further confirms that the reaction occurs between the quasi-metallic Na in the HC-1300 electrode and ethanol


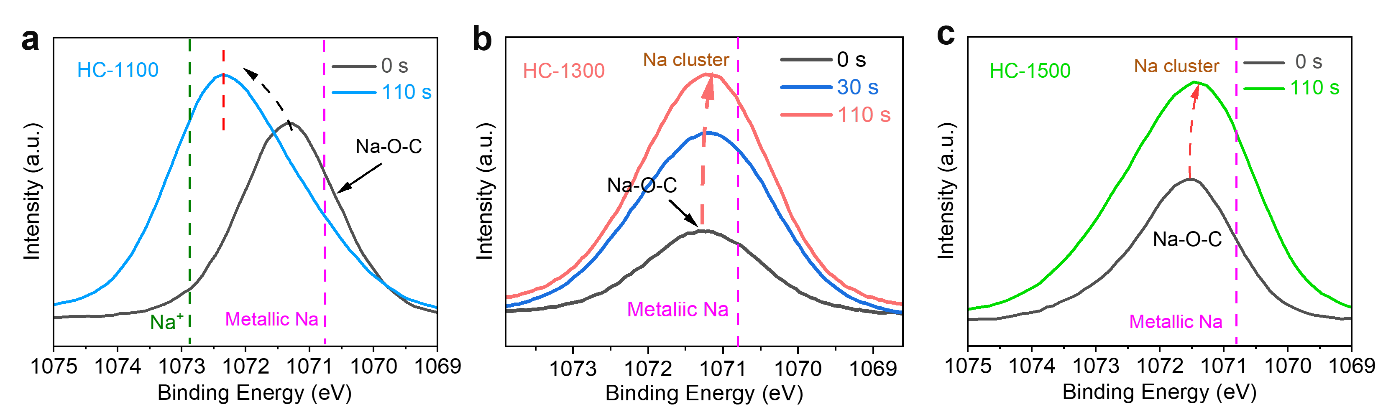


**Fig. S21** XPS spectra of Na 1s at different etching times for three fully sodiated HCs. A characteristic Na–O–C peak at 1071.4 eV observed in the unsputtered samples can be attributed to Na species present in the solid electrolyte interphase (SEI) [S17]


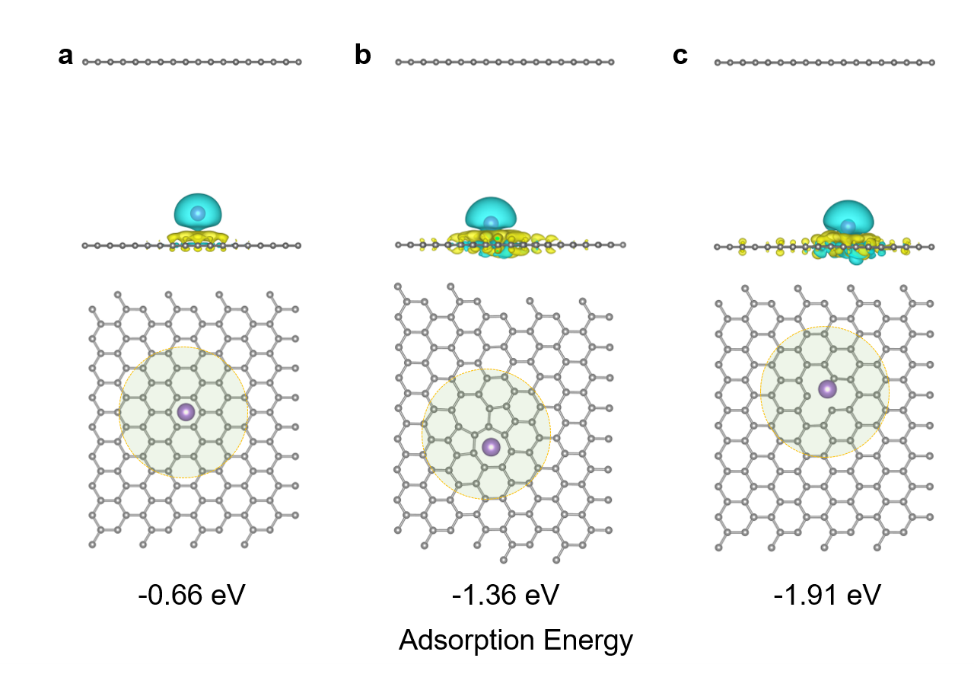


**Fig. S22** Adsorption of Na ions at the defective sites within the cavities. **a-c** A graphitic wall with no defect (**a**), a 5-7-7-5 defect (**b**), and a point defect (**c**). Because of the increased adsorption energy for Na adsorption, these internal defects can also serve as additional energy favorable nucleation sites for Na-cluster formation within cavities. Pink: Na; grey: C; yellow: electron rich; cyan: electron deficient; isosurface: 0.0005


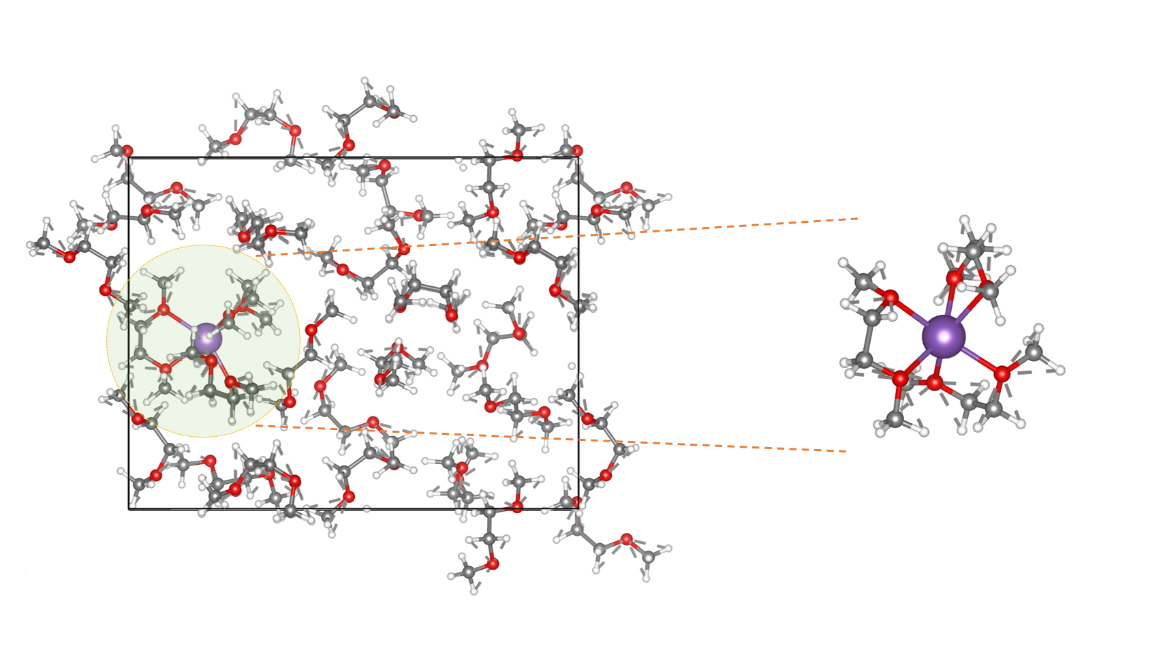


**Fig. S23** Solvation structure of Na ions with DME molecules, obtained by AIMD simulations. Pink: Na; grey: C; white: H; red: O.


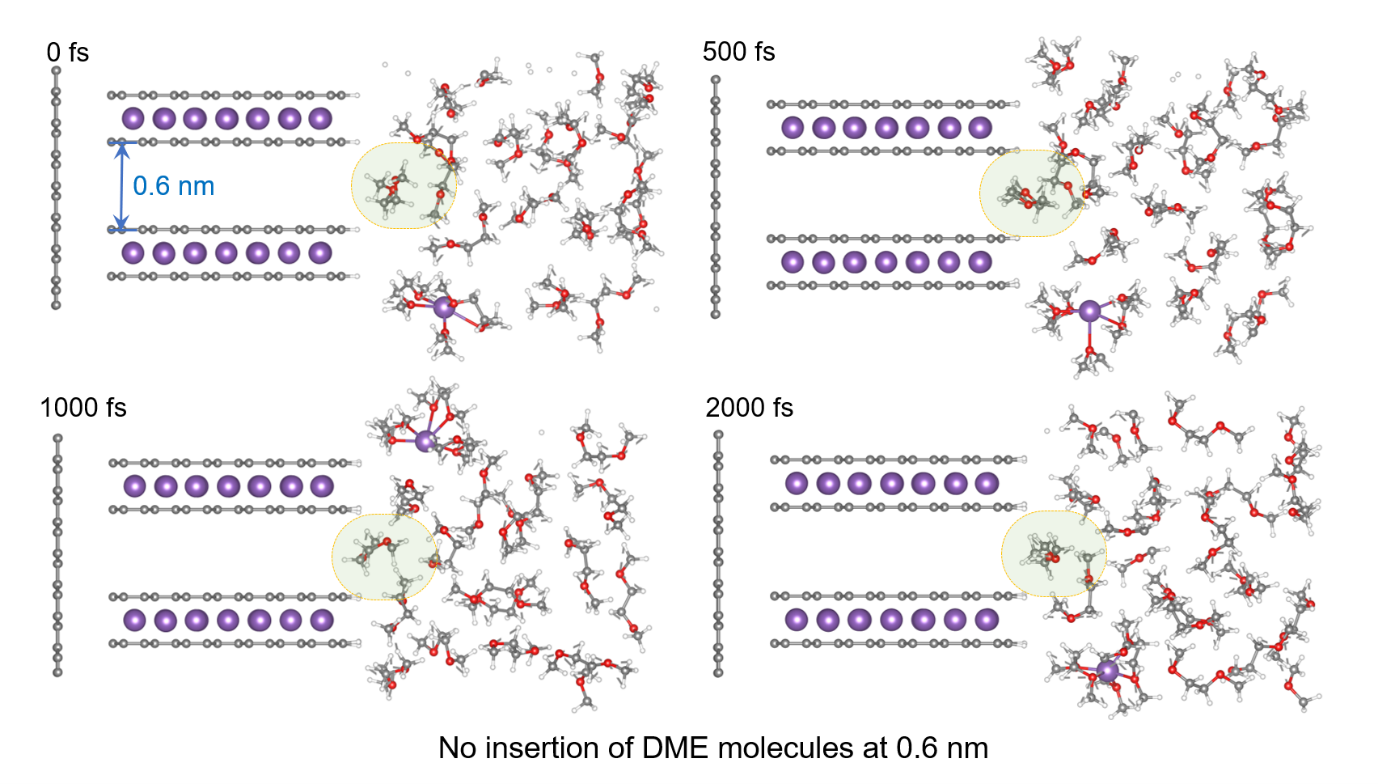


**Fig. S24** Snapshots of AIMD simulations at 0, 500, 1000, and 2000 fs, revealing no intercalation of individual DME molecules into graphitic layers with a *d*-spacing of 0.6 nm. Pink: Na; grey: C; white: H; red: O


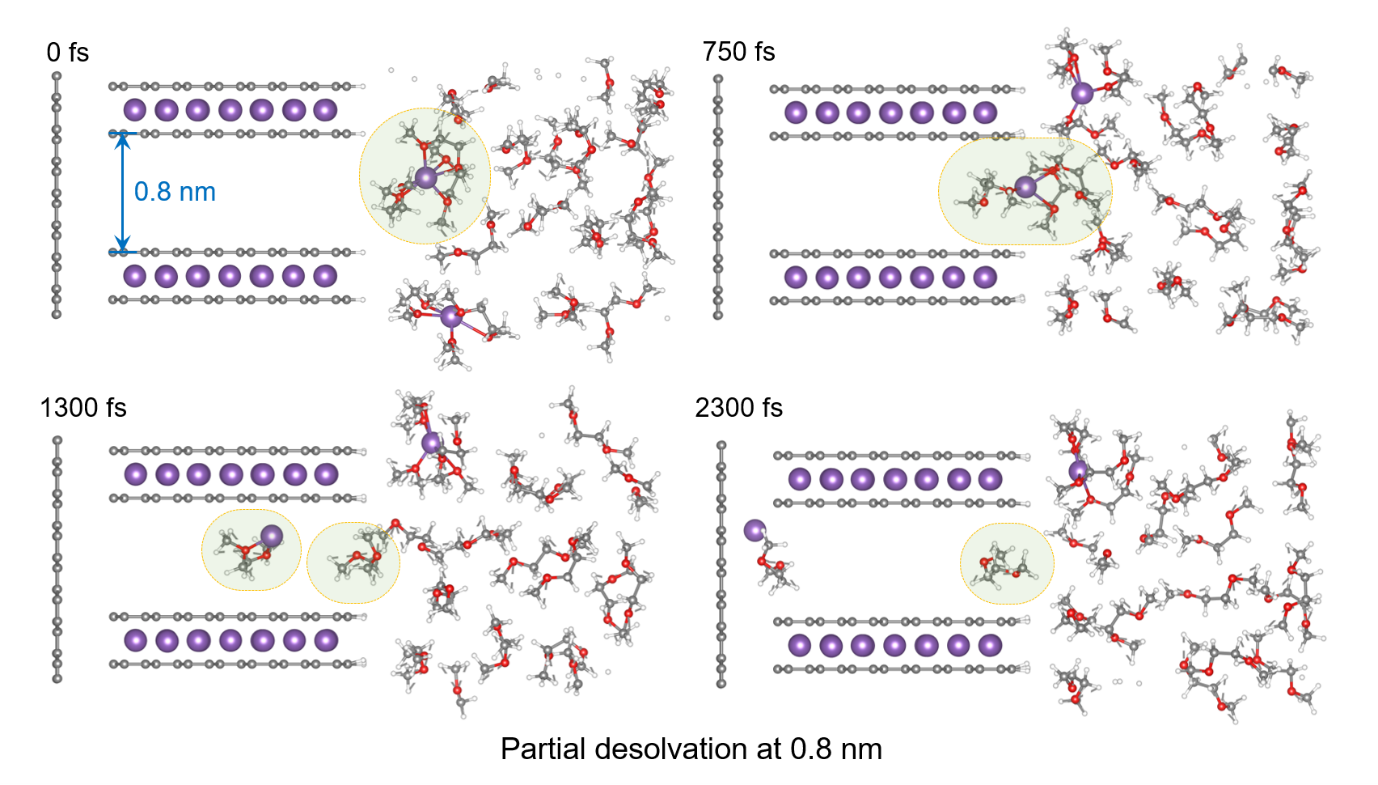


**Fig. S25** Snapshots of AIMD simulations at 0, 750, 1300, and 2300 fs, revealing partial desolvation of Na ion and intercalation of individual DME molecules into graphitic layers with a *d*-spacing of 0.8 nm. Pink: Na; grey: C; white: H; red: O


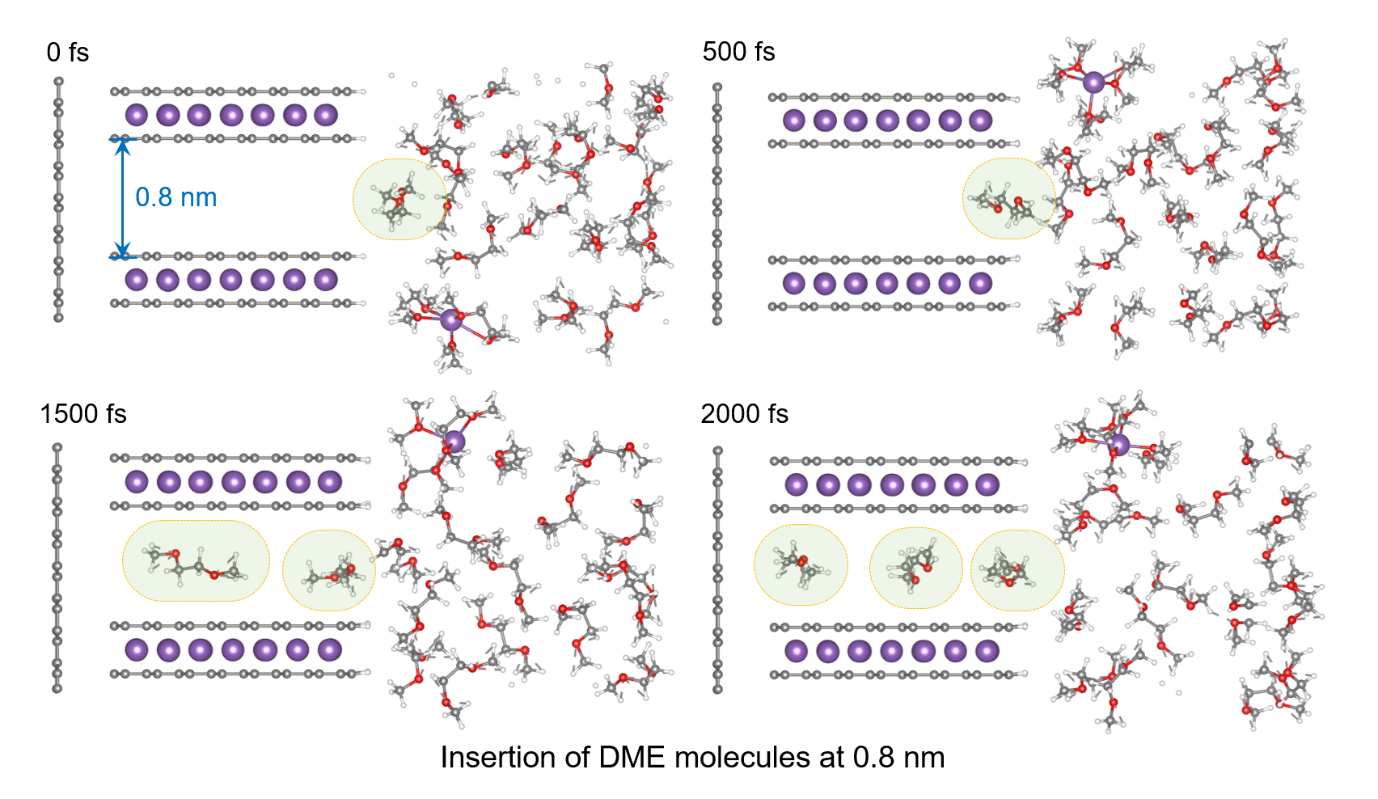


**Fig. S26** Snapshots of AIMD simulations at 0, 500, 1500, and 2000 fs, revealing the intercalation of individual DME molecules into graphitic layers with a *d*-spacing of 0.8 nm. Pink: Na; grey: C; white: H; red: O


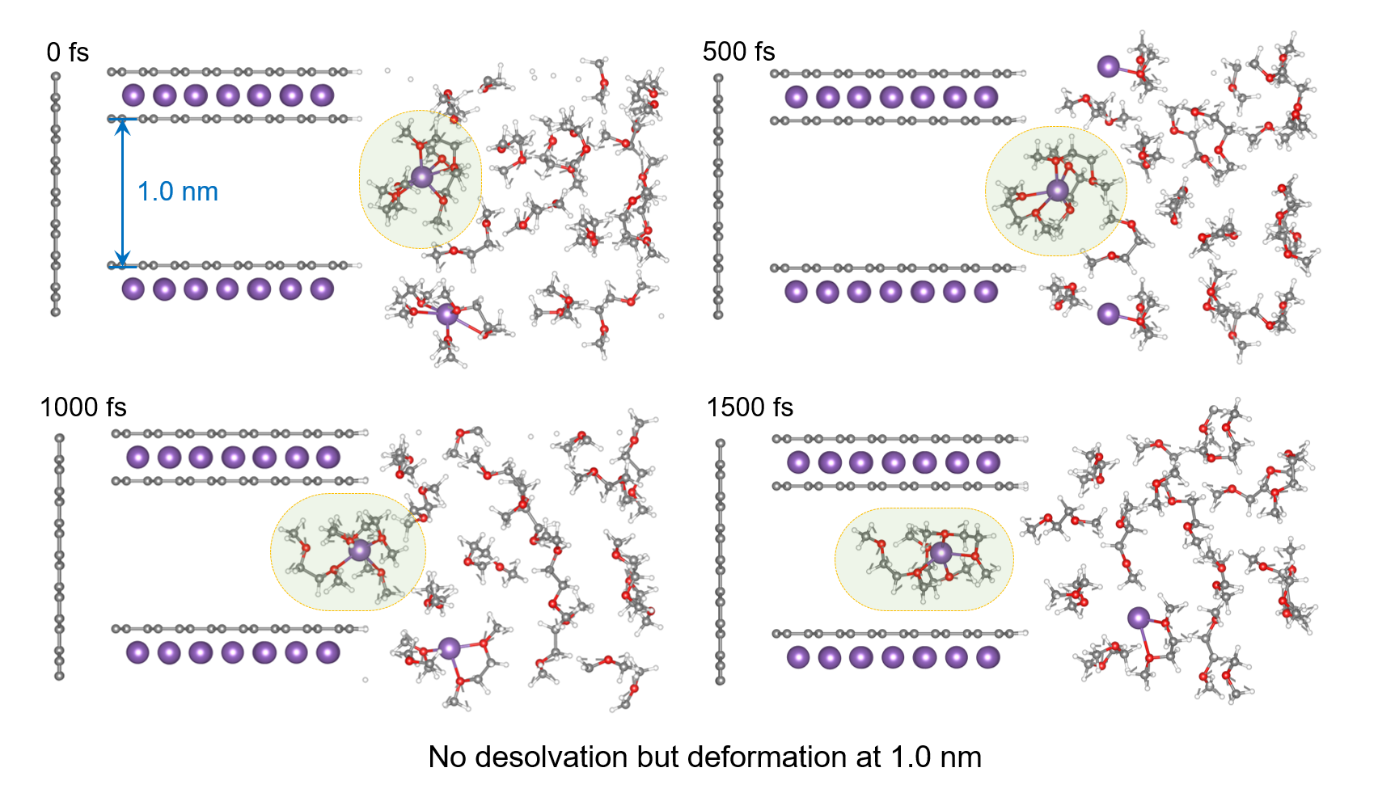


**Fig. S27** Snapshots of AIMD simulations at 0, 500, 1000, and 1500 fs, revealing deformation of a solvated Na ion into graphitic layers with a *d*-spacing of 1.0 nm. Pink: Na; grey: C; white: H; red: O


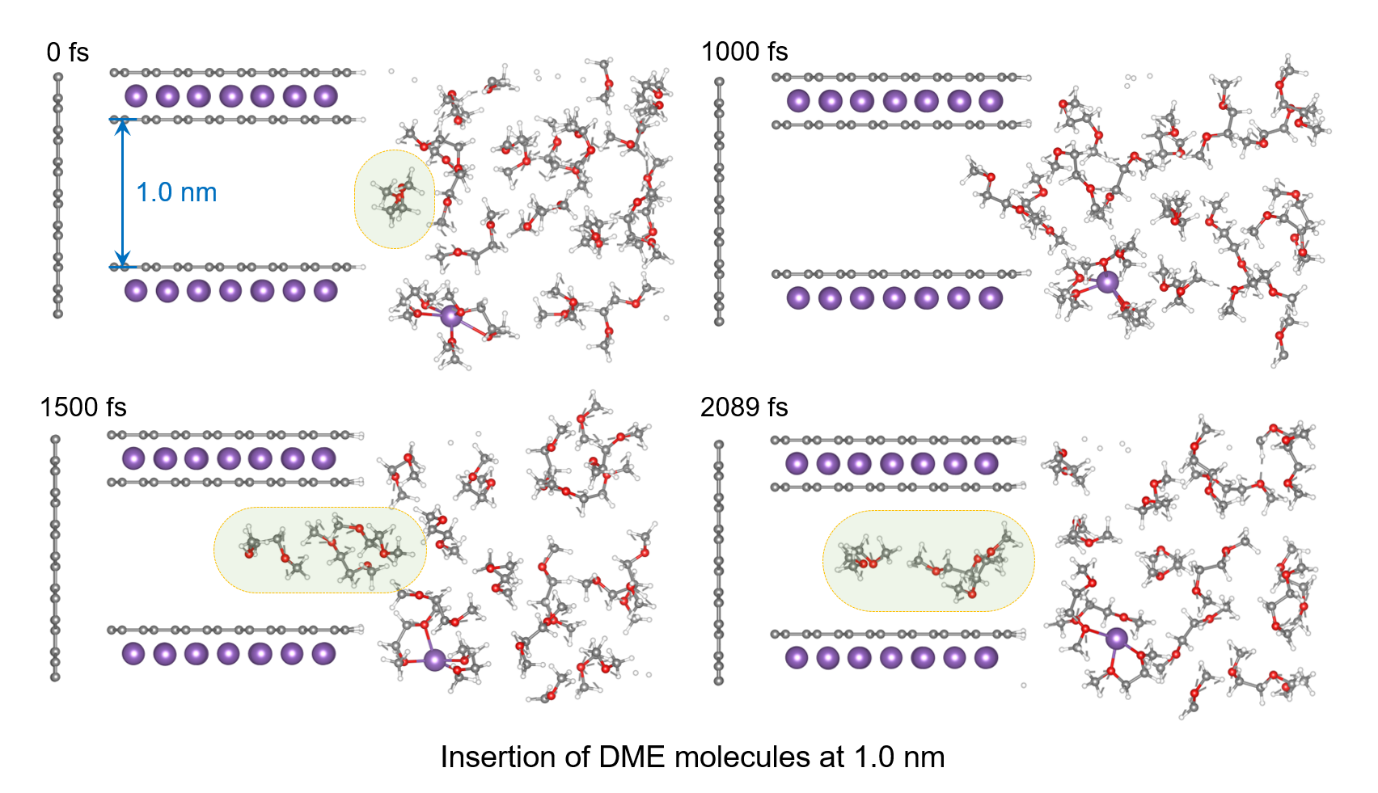


**Fig. S28** Snapshots of AIMD simulations at 0, 1000, 1500, and 2089 fs, revealing the intercalation of individual DME molecules into graphitic layers with a *d*-spacing of 1.0 nm. Pink: Na; grey: C; white: H; red: O


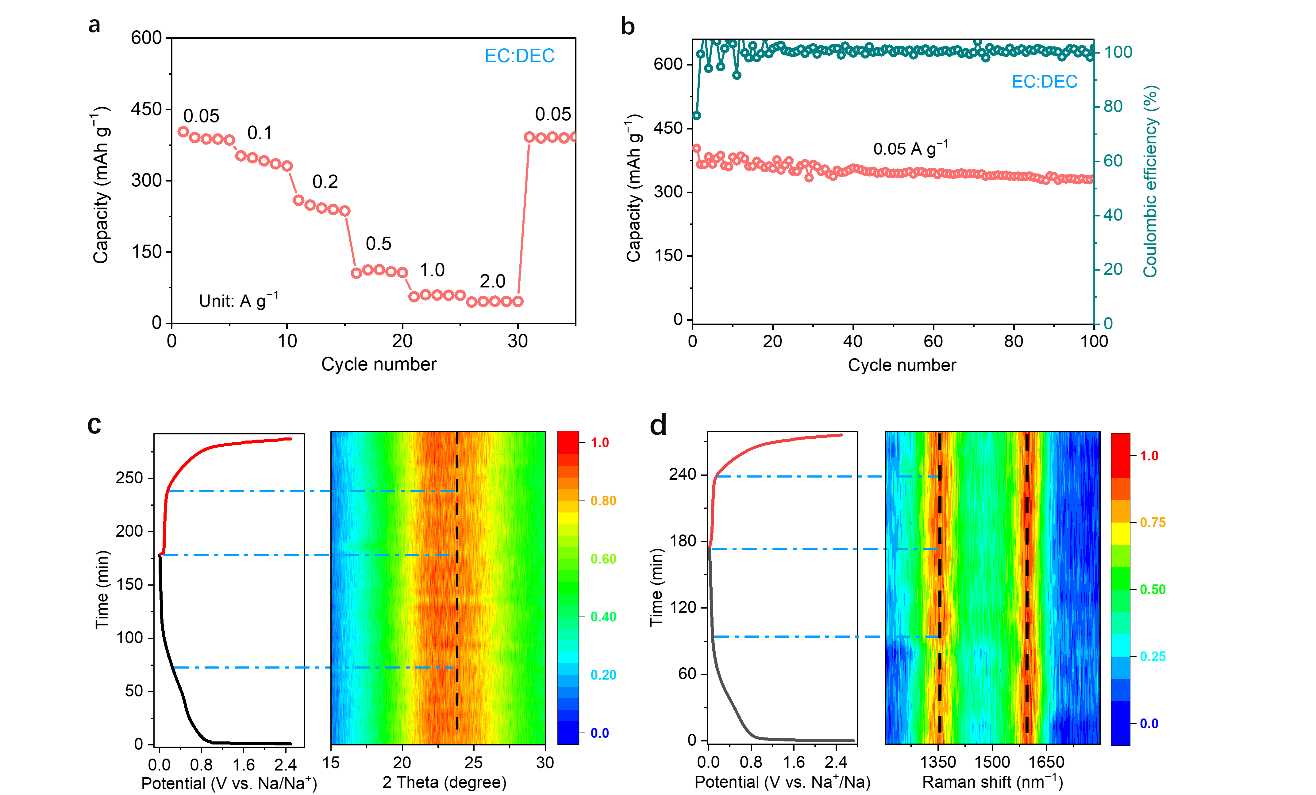


**Fig. S29** Characterization of the HC-1300 electrode in the ester-based (EC/DEC) electrolyte. **a** Rate capability of the HC-1300 electrode at different current densities. **b** Cycling performance of the HC-1300 electrode at 50 mA g^–1^. **c**,**d** Operando XRD patterns (**c**) and Raman spectra (**d**) of the HC-1300 electrode in the ester-based electrolyte during the initial charge-discharge process

The electrochemical performance of HC-1300 was also evaluated in an ester-based electrolyte consisting of 1 M NaPF₆ in ethylene carbonate (EC)/diethyl carbonate (DEC) (1:1 vol%). As shown in Figs. S29a,b, the HC-1300 electrode delivers a capacity of 403 mAh g^–1^ at 50 mA g^–1^, which is lower than the ~500 mAh g^–1^ obtained for the same electrode in the ether-based electrolyte. In addition, as the current density increases from 50 to 2000 mA g^–1^, the HC-1300 electrode in the ester-based electrolyte retains only 11.8% of its initial capacity at 2000 mA g^–1^. After 100 cycles at 50 mA g^–1^, the capacity retention is 82.3%. Overall, the electrochemical performance in the ester-based electrolyte is consistently inferior to that observed in the ether-based electrolyte.

Operando XRD and Raman measurements were performed further. It can be seen in Fig. S29c that, during the charge-discharge process, especially in the plateau region, the position and intensity of the (002) peak of HC-1300 in the ester-based electrolyte remain nearly unchanged. This indicates that the sodiation process has only a minimal effect on the interlayer structure at the closed-pore orifice, suggesting that little intercalation occurs. These results imply that pre-desolvation is more difficult for solvated Na⁺ in EC/DEC solvents, which is consistent with previous studies.[S18, S19] It further suggests that the highly coupled “intercalation–pore-filling” storage mechanism is largely suppressed in the ester-based electrolyte. Instead, pore filling is more likely to occur only after Na⁺ passes through relatively large orifices (>0.4 nm).

Moreover, Fig. S29d indicates that the D band (~1350 cm⁻¹) of HC-1300 in the ester-based electrolyte does not disappear in the plateau region, in contrast to the behavior observed for the same electrode in the ether-based electrolyte (Fig. 3h). This suggests that some internal graphitic defects remain uncovered during sodiation because closed pores with smaller orifices cannot be effectively accessed by Na⁺ and therefore cannot accommodate deposited Na clusters. Taken together, while the proposed mechanism remains valid, these operando characterizations explain the lower capacity and decreased rate capability observed for HC-1300 in the ester-based electrolyte compared with those obtained in the ether-based electrolyte.


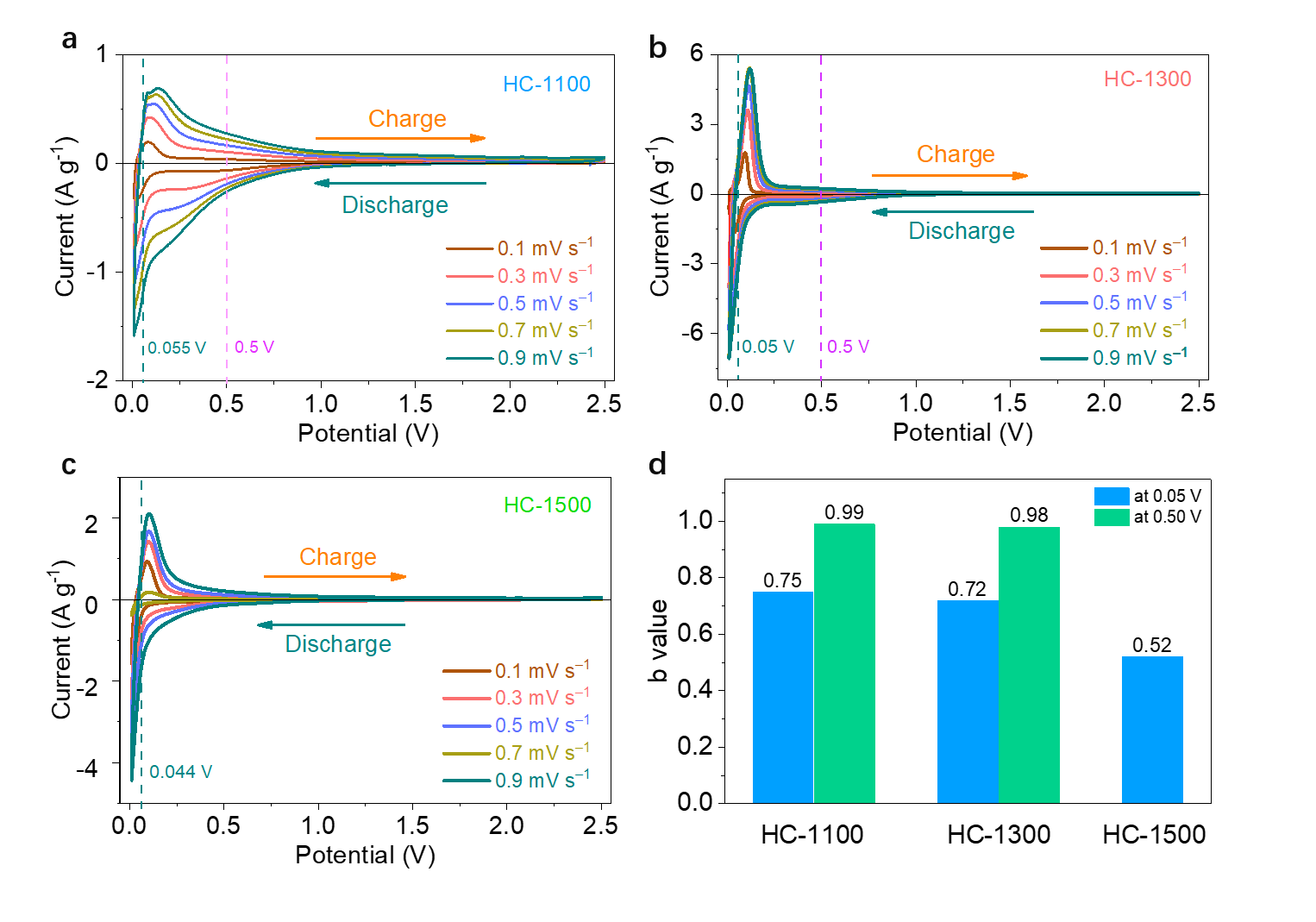


**Fig. S30** Cyclic voltammetry (CV) results. **a-c** CV curves for HC-1100 (**a**), HC-1300 (**b**), and HC-1500 (**c**) electrodes at different scan rates, ranging from 0.1 mV s^–1^ to 0.9 mV s^–1^. **d** The comparison of *b* values, indicating the changes of kinetics


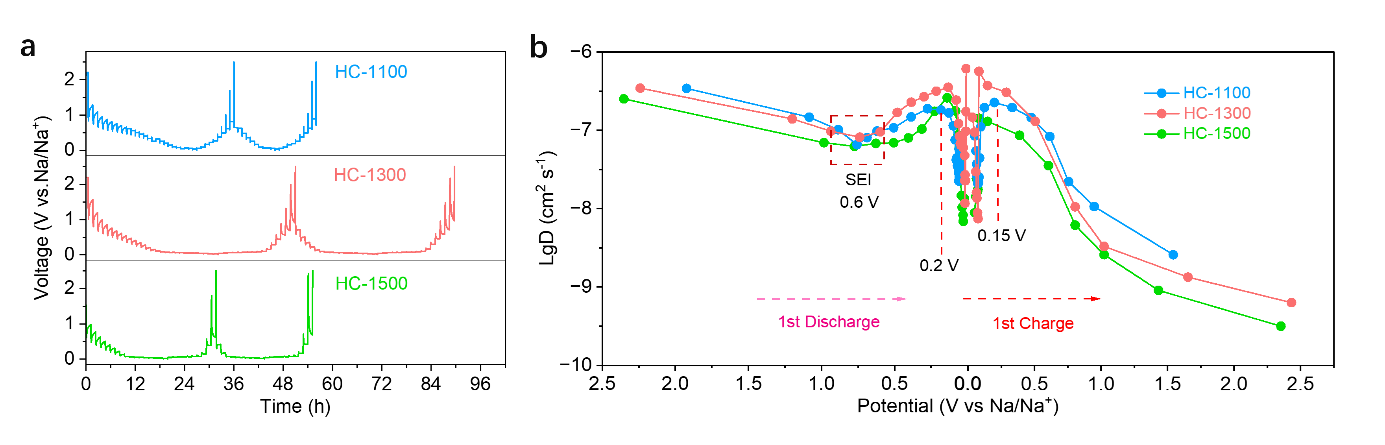


**Fig. S31** Na-ion diffusion coefficients calculated based on GITT profiles. For the GITT tests, the cell were discharged/charged at 100 mA g^−1^ with a current pulse duration of 10 min and interval time of 60 min. The ion diffusion coefficient in three HC electrodes can be employed by the followed Equation [S20]:

$$D_{Na^{+}}=\frac{4}{\pi\Delta\tau}(\frac{m_{B}V_{M}}{M_{B}S})^{2}(\frac{\Delta E_{s}}{\Delta E_{\tau}})^{2}$$

Where $m_{B}$ is the mass of the active materials in the electrode, $M_{B}$ and $V_{M}$ is the molar mass and molar volume of the electrode, and *S* is the geometric area of the electrode. Obviously, the $M_{B}/V_{M}$ can be obtained from the density of electrode materials.


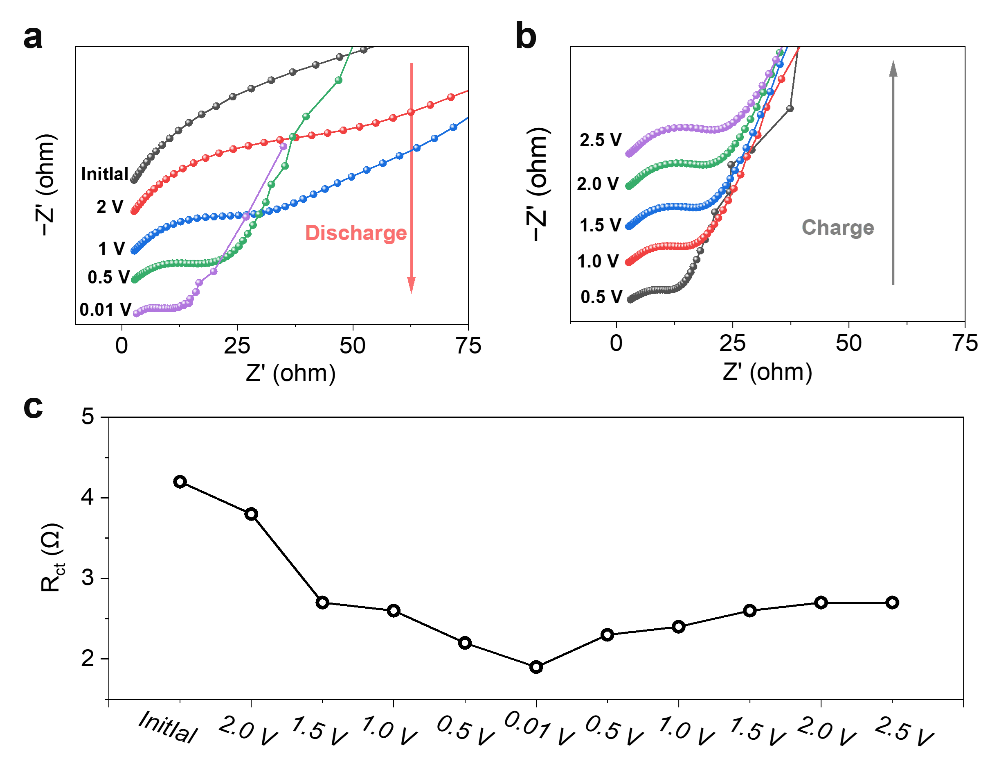


**Fig. S32** In-situ EIS profiles of HC-1300 electrodes with the ether-based electrolyte in three-electrode system during the first galvanostatic process at 150 mA g^−1^. **a**, Sodiation process. **b**, Desodiation process. **c**, *R*_ct_ as a function of potential during the first sodiation and desodiation processes


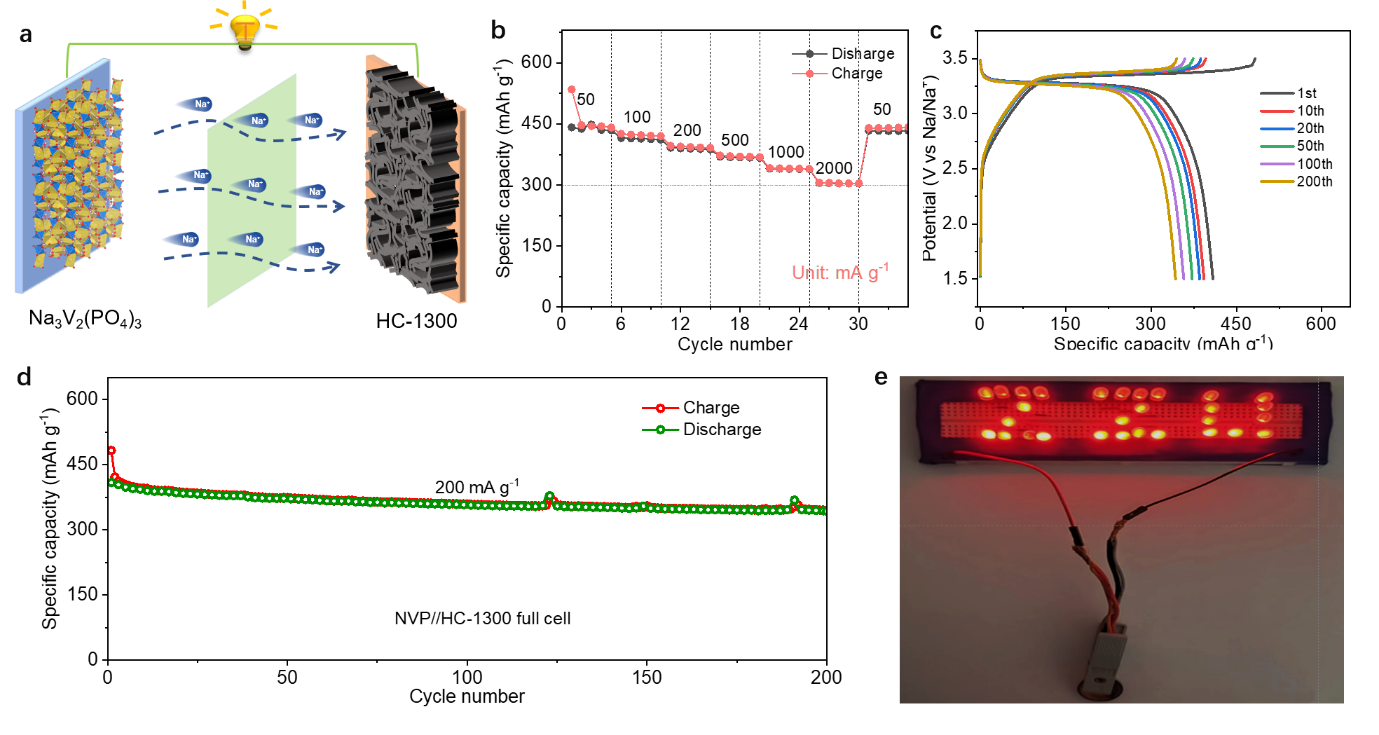


**Fig. S33** Electrochemical performance of coin-type full cells. **a** The coin full cells with NVP as cathode and HC-1300 as anode were assembled. **b-d** Rate capability (**b**), Charge-discharge curves (**c**), and cycling performance (**d**) of the coin-type full cells. **e** A photograph showing the practical potential of NVP||HC-1300 full cell by illuminating the “ZZU” patterned 29 red light-emitting diodes (LEDs)

**Table S1** Parameter values of the electrodes

| Parameter | Cathode | Anode |
| --- | --- | --- |
| Active material | 90.0% | 90.0% |
| Conductive agent | 5.0% | 5.0% |
| Binder | 5.0% | 5.0% |

The active materials of the cathode and anode are NFPP and HC-1300, respectively.

**Table S2** Parameter values of SIBs

| Parameter | Value for the HC||NFPP pouch battery |
| --- | --- |
| Cathode mass loading | 25.5 mg cm^−2^ |
| Anode mass loading | 6.3 mg cm^−2^ |
| P/N ratio | 1.2 |
| Electrolyte (1M NaPF_6_ DME) | 6.1 g |
| Pouch battery weight | 32.2 g |
| Capacity | 1.5 Ah |
| Energy | 4.8 Wh |
| Energy density | 147.4 Wh kg^−1^ |

**Supporting Movies**

**Movie S1** The reaction phenomenon between a fully-discharged HC-1100 electrode and ethanol.

**Movie S2** The reaction phenomenon between a fully-discharged HC-1300 electrode and ethanol.

**Movie S3** The reaction phenomenon between a fully-discharged HC-1500 electrode and ethanol.

**Movie S4** AIMD simulations of a solvated Na ion inserted into graphitic layers with a *d*-spacing of 0.6 nm, revealing the pre-desolvation process. Pink: Na; grey: C; white: H; red: O.

**Movie S5** AIMD simulations of a solvated Na ion inserted into graphitic layers with a *d*-spacing of 0.8 nm, revealing the partial desolvation of Na ion and intercalation of individual DME molecules. Pink: Na; grey: C; white: H; red: O.

**Movie S6** AIMD simulations of a solvated Na ion inserted into graphitic layers with a *d*-spacing of 1.0 nm, revealing the deformation of a solvated Na ion. Pink: Na; grey: C; white: H; red: O.

**Supplementary References**

1. H. Sun, Q. Zhang, F. Yuan, D. Zhang, Z. Li et al., Unraveling the effect of carbon morphology evolution in hard carbons on sodium storage performance. Inorg. Chem. Front. **10**(22), 6547–6556 (2023). <https://doi.org/10.1039/D3QI01497E>
2. L. Kitsu Iglesias, E.N. Antonio, T.D. Martinez, L. Zhang, Z. Zhuo et al., Revealing the sodium storage mechanisms in hard carbon pores. Adv. Energy Mater. **13**(44), 2302171 (2023). <https://doi.org/10.1002/aenm.202302171>
3. D.A. Stevens, J.R. Dahn, An *in situ* small-angle X-ray scattering study of sodium insertion into a nanoporous carbon anode material within an operating electrochemical cell. J. Electrochem. Soc. **147**(12), 4428 (2000). <https://doi.org/10.1149/1.1394081>
4. Z. Tang, S. Zhou, P. Wu, H. Wang, Y. Huang et al., Engineering surface oxygenated functionalities on commercial hard carbon toward superior sodium storage. Chem. Eng. J. **441**, 135899 (2022). <https://doi.org/10.1016/j.cej.2022.135899>
5. X. Feng, F. Wu, Y. Li, Y. Fu, Y. Li et al., Critical role of ultra-microporous tunnel structure within hard carbon in boosting sodium-ion storage. Adv. Mater. **38**(1), e01779 (2026). <https://doi.org/10.1002/adma.202501779>
6. Z. Li, Y. Chen, Z. Jian, H. Jiang, J.J. Razink et al., Defective hard carbon anode for Na-ion batteries. Chem. Mater. **30**(14), 4536–4542 (2018). <https://doi.org/10.1021/acs.chemmater.8b00645>
7. R. Zhou, F. Zhou, Y. Huang, Y. Zhao, C. Bao et al., Zinc−Ion−Assisted catalytic synthesis of hard carbon with superior plateau capacity and ICE for Sodium−Ion batteries. Adv. Funct. Mater. e27331 (2026). <https://doi.org/10.1002/adfm.202527331>
8. S. Xiao, J. Xu, Engineering of closed pores and transport channels in polymer-derived hard carbon *via* chemical vapor deposition for sodium-ion batteries. ACS Nano **20**(14), 11096–11107 (2026). <https://doi.org/10.1021/acsnano.5c21256>
9. X. Li, J. Zhang, J. Zhang, L. Guo, H. Zhang et al., C60 to modulate the closed pore structures of hard carbon for high-performance sodium-ion batteries. ACS Nano **19**(15), 14829–14838 (2025). <https://doi.org/10.1021/acsnano.4c18421>
10. Z. Tang, R. Zhang, H. Wang, S. Zhou, Z. Pan et al., Revealing the closed pore formation of waste wood-derived hard carbon for advanced sodium-ion battery. Nat. Commun. **14**(1), 6024 (2023). <https://doi.org/10.1038/s41467-023-39637-5>
11. F. Chen, Y. Di, Q. Su, D. Xu, Y. Zhang et al., Vanadium-modified hard carbon spheres with sufficient pseudographitic domains as high-performance anode for sodium-ion batteries. Carbon Energy **5**(2), e191 (2023). <https://doi.org/10.1002/cey2.191>
12. C. Qiu, A. Li, D. Qiu, Y. Wu, Z. Jiang et al., One-step construction of closed pores enabling high plateau capacity hard carbon anodes for sodium-ion batteries: closed-pore formation and energy storage mechanisms. ACS Nano **18**(18), 11941–11954 (2024). <https://doi.org/10.1021/acsnano.4c02046>
13. C. Chen, Y. Tian, R. Ren, S. Duan, D. Wang et al., Regulating pores and carbonyl groups of biomass-derived hard carbon for enhanced sodium storage. Adv. Sci. **12**(40), e10328 (2025). <https://doi.org/10.1002/advs.202510328>
14. Y. Li, W. Wang, B. Wang, P. Wang, Z. He et al., Dual-beam facilitated oxygen manipulation in hard carbon for improved sodium storage. Nano Res. **19**(1), 94908317 (2026). <https://doi.org/10.26599/nr.2026.94908317>
15. S. Alvin, D. Yoon, C. Chandra, H.S. Cahyadi, J.-H. Park et al., Revealing sodium ion storage mechanism in hard carbon. Carbon **145**, 67–81 (2019). <https://doi.org/10.1016/j.carbon.2018.12.112>
16. Y.-E. Zhu, L. Yang, X. Zhou, F. Li, J. Wei et al., Boosting the rate capability of hard carbon with an ether-based electrolyte for sodium ion batteries. J. Mater. Chem. A **5**(20), 9528–9532 (2017). <https://doi.org/10.1039/C7TA02515G>
17. Z. Wang, X. Feng, Y. Bai, H. Yang, R. Dong et al., Probing the energy storage mechanism of quasi-metallic Na in hard carbon for sodium-ion batteries. Adv. Energy Mater. **11**(11), 2003854 (2021). <https://doi.org/10.1002/aenm.202003854>
18. F. Cheng, J. Hu, W. Zhang, B. Guo, P. Yu et al., Reviving ether-based electrolytes for sodium-ion batteries. Energy Environ. Sci. **18**(14), 6874–6898 (2025).
19. G. Liu, Z. Wang, H. Yuan, C. Yan, R. Hao et al., Deciphering electrolyte dominated Na^+^ storage mechanisms in hard carbon anodes for sodium-ion batteries. Adv. Sci. **10**(36), 2305414 (2023). <https://doi.org/10.1002/advs.202305414>
20. J. Zhu, J. Fan, T. Cheng, M. Cao, Z. Sun et al., Bilayer nanosheets of unusual stoichiometric bismuth oxychloride for potassium ion storage and CO_2_ reduction. Nano Energy **75**, 104939 (2020). <https://doi.org/10.1016/j.nanoen.2020.104939>
